# Identification of an Epi-metabolic dependency on *EHMT2*/G9a in T-cell acute lymphoblastic leukemia

**DOI:** 10.1038/s41419-022-05002-5

**Published:** 2022-06-17

**Authors:** Anna Montanaro, Samuel Kitara, Elisa Cerretani, Matteo Marchesini, Chiara Rompietti, Luca Pagliaro, Andrea Gherli, Angela Su, Maria Laura Minchillo, Mariafrancesca Caputi, Rodanthi Fioretzaki, Bruno Lorusso, Linda Ross, Gabriela Alexe, Elena Masselli, Marina Marozzi, Federica Maria Angela Rizzi, Roberta La Starza, Cristina Mecucci, Yan Xiong, Jian Jin, Angela Falco, Birgit Knoechel, Franco Aversa, Olivia Candini, Federico Quaini, Paolo Sportoletti, Kimberly Stegmaier, Giovanni Roti

**Affiliations:** 1 Department of Medicine and Surgery, University of Parma, Parma, 43126 Italy; 2 Department of Pediatric Oncology, Dana-Farber Cancer Institute, Harvard Medical School, Boston, MA 02215 USA; 3 Department of Medical Science, University of Ferrara, Ferrara, 44121 Italy; 4 IRCCS Istituto Romagnolo per lo Studio dei Tumori “Dino Amadori” IRST (S.r.l.), Meldola, 47014 Italy; 5 Department of Medicine, Hematology and Clinical Immunology, University of Perugia, Perugia, 06123 Italy; 6 Azienda-Ospedaliera di Parma, Hematology and BMT Unit, Parma, 43126 Italy; 7 National Institute for Biostructures and Biosystems (I.N.B.B.), Rome, Italy; 8 Mount Sinai Center for Therapeutics Discovery, Departments of Pharmacological Sciences and Oncological Sciences, Tisch Cancer Institute, Icahn School of Medicine at Mount Sinai, New York, NY 10029 USA; 9 Division of Hematology/Oncology, Boston Children’s Hospital, Boston, MA 02215 USA; 10 Rigenerand S.r.l., Medolla, Modena, 41036 Italy; 11 The Broad Institute, Cambridge, MA 02142 USA

**Keywords:** Translational research, Cancer metabolism, Acute lymphocytic leukaemia

## Abstract

Genomic studies have identified recurrent somatic alterations in genes involved in DNA methylation and post-translational histone modifications in acute lymphoblastic leukemia (ALL), suggesting new opportunities for therapeutic interventions. In this study, we identified G9a/*EHMT2* as a potential target in T-ALL through the intersection of epigenome-centered shRNA and chemical screens. We subsequently validated G9a with low-throughput CRISPR-Cas9-based studies targeting the catalytic G9a SET-domain and the testing of G9a chemical inhibitors in vitro, 3D, and in vivo T-ALL models. Mechanistically we determined that G9a repression promotes lysosomal biogenesis and autophagic degradation associated with the suppression of sestrin2 (*SESN2)* and inhibition of glycogen synthase kinase-3 (GSK-3), suggesting that in T-ALL glycolytic dependent pathways are at least in part under epigenetic control. Thus, targeting G9a represents a strategy to exhaust the metabolic requirement of T-ALL cells.

## Introduction

The identification of genes involved in DNA methylation and post-translational histone modifications in hematologic malignancies offers an opportunity for novel therapeutic intervention, geared towards reversing or modulating epigenetic events underpinning the leukemic state. Recently, several molecules have entered early clinical phase testing in hematologic neoplasms. Tazemetostat, the histone methyltransferase (HMT) inhibitor of enhancer of zeste homologue 2 (*EZH2*) [1], is currently in a single agent open-label, phase 1/2 trial in patients with B-cell lymphomas and in combination with prednisolone in diffuse large B-cell lymphoma (DLBCL) (NCT01897571). Pinometostat (EPZ-5676), an inhibitor of the disruptor of telomeric silencing-1 gene (*DOT1L*), has been assessed in phase 1 dose-escalation studies in pediatric and adult patients with relapsed and refractory AML and ALL [2, 3]. Similarly, pharmacologic inhibition of menin-lysine methyltransferase 2A (*KMT2A*) binding proved to be an effective antileukemic strategy in preclinical models of *KMT2A* related leukemias [4] and supported clinical trials evaluating menin inhibitors as targeted therapies in acute leukemia [5].

The G9a/GLP complex represents an additional, druggable methyltransferase target. In fact, several groups have developed inhibitors targeting G9a and GLP with high specificity [6–9]. G9a and GLP are conserved protein lysine methyltransferases that contain a Su(Var), enhancer of zeste, trithorax (SET) domain. G9a localizes in euchromatin regions and regulates gene expression and chromosome structure through de novo mono- and di- methylation of histone H3 lysine 9 (H3K9me1/2). Di- and trimethylation of lysine 9 of histone H3 (H3K9) in gene promoters have been associated with transcriptional repression in several disease models [10, 11]. *EHMT2*, which encodes for the G9a protein, has emerged as a potential tumor biomarker of aggressive cancers [11, 12].

In this manuscript, the intersection of multiple “omics” approaches identified G9a as a therapeutic target in T-cell acute lymphoblastic leukemia (T-ALL), an aggressive neoplastic disorder of lymphoblasts committed to the T-cell lineage in need of new treatment modalities, particularly in relapsed/refractory cases [13]. We determined that G9a suppression inhibits the metabolic sensor sestrin2 (*SESN2*), promotes lysosomal biogenesis, autophagic degradation and, ultimately leading to the inactivation of Glycogen Synthase Kinase-3 (GSK-3) with impaired glycogen metabolism.

## Results

### G9a is a potential therapeutic target in T-ALL

**Fig. 1:**
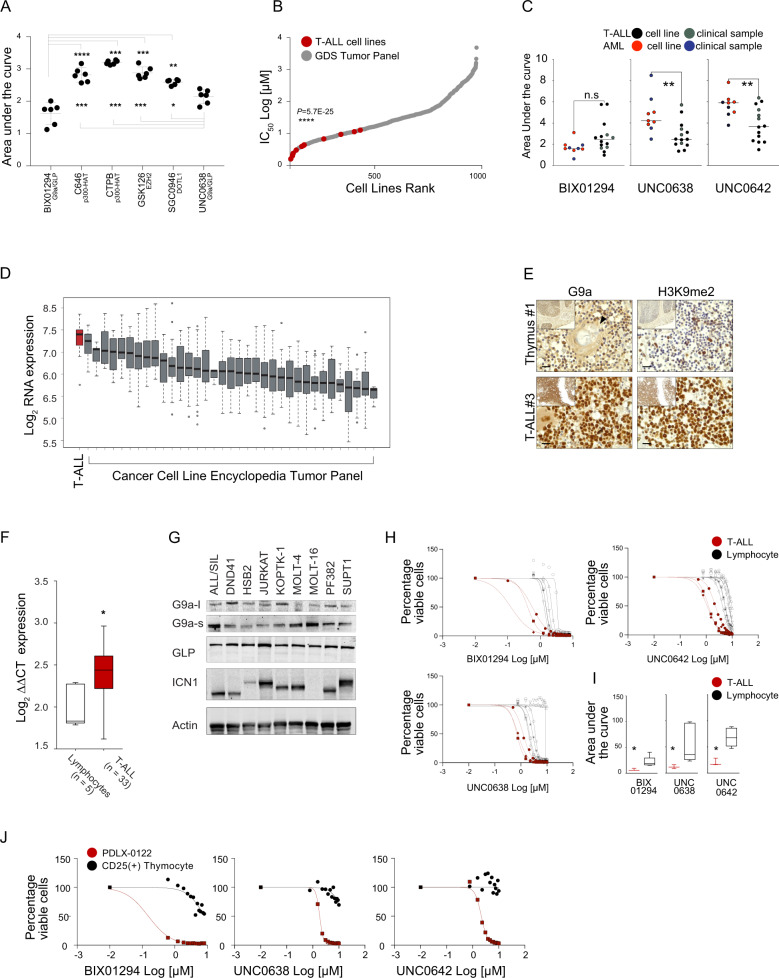
T-ALL cells are dependent on *EHMT2*. **A** Effect of epigenetic modulators BIX01294, C646, CTPB, GSK126, SGC0946, and UNC0638 in T-ALL cell lines (ALL/SIL, CCRF-CEM, DND41, HPB-ALL, HSB2, and PEER). BIX01294 is a diazepin-quinazolin-amine derivative that selectively inhibits the H3K9 di-methylation activity of G9a, and to a lesser extent GLP, without competing for the S-adenosyl-methionine (SAM) cofactor; C646 is a selective small-molecule inhibitor of histone acetyltransferase p300; CTPB is an amide derivative that selectively activates the histone acetyltransferase (HAT) p300; GSK126 is a EZH2 methyltransferase inhibitor; SGC0946 is a highly potent and selective DOT1L methyltransferase inhibitor; UNC0638 is a substrate-competitive small-molecule inhibitor with equal potency for G9a and GLP in cell-based assays. The scatter dot plot represents the effect of small molecules on cellular viability calculated using the area under the curve (AUC) model of log transformed dose-response data using GraphPad V7. A lower AUC corresponds to a greater sensitivity. Statistical significance among groups for treated vs. vehicle (DMSO) (*P ≤ 0.05, **P ≤ 0.01, ***P ≤ 0.001, ****P ≤ 0.0001) was determined by one-way ANOVA using Bonferroni’s correction for multiple comparison testing. **B** Graph showing response to the G9a inhibitor, UNC0638, in over 900 cancer cell lines screened as part of the Genomics of Drug Sensitivity in Cancer Project (GDS). T-ALL cell lines are indicated in red, non-T-ALL cell lines in grey. Statistical significance among groups (****P ≤ 0.0001) was determined by a non-parametric *t*-test (Mann–Whitney). **C** Effect of epigenetic modulators BIX01294, UNC0638, UNC0642 in T-ALL (*n* = 10), AML (*n* = 5) cell lines and primary patients T-ALL cells (*n* = 4) and primary patients AML cells (*n* = 4) samples. The scatter dot plot represents the effect of small molecules on cellular viability calculated using the area under the curve (AUC) model of log transformed dose-response (BIX01294 = 0 < [*X*] < 8 μM; UNC038 = 0 < [*X*] < 10 μM; UNC042 = 0 < [*X*] < 10 μM) data using GraphPad V7. A lower AUC corresponds to a greater sensitivity. Statistical significance among groups (**P ≤ 0.01) was determined by a non-parametric *t*-test (Mann–Whitney). **D** *EHMT2* expression levels in 36 cancer types (1036 cancer cell lines). Data were obtained from the Cancer Cell Line Encyclopedia [16]. A red bar represents the T-ALL cell lines. **E** G9a and H3K9me2 expression in human thymus and T-ALL lymphoblasts. Formalin-fixed, paraffin embedded tissue sections were stained with anti-G9a and anti-H3K9me2. Scale bars, 20 μm. Hassall’s corpuscles are indicated by arrowheads. **F** *EHMT2* expression levels in T cells lymphocytes (*n* = 5) or in T-ALL lymphoblasts (*n* = 33). The line in the box-and-whisker diagram represents the Log_2_ *EHMT2* median expression calculated according to the ΔΔCT method. The upper edge (hinge) of the box indicates the 75th percentile of the data, and the lower hinge the 25th percentile. The end of the vertical line represents the minimum and the maximum data values. Statistical significance among groups (*P ≤ 0.05) was determined by a non-parametric *t*-test (Mann–Whitney). **G** Western blot showing expression of G9a (l long isoform, s short isoform), GLP, and ICN1 in a panel of T-ALL cell lines. Actin was used as a loading control. **H** Effect of G9a inhibitors BIX01294, UNC0638, and UNC0642 in primary T-ALL blasts (*n* = 3) or isolated T cells (*n* = 6). Cells were grown at increasing concentrations of G9a/GLP inhibitors (BIX01294, UNC0638, and UNC0642) and viability evaluated at day 3 by an ATP-based assay and plotted as the percentage of viable cells relative to a DMSO control. Shown is the mean ± standard deviation (SD) of a minimum of two replicates. **I** Effect of the G9a inhibitors BIX01294, UNC0638, and UNC0642 in primary T-ALL cells (*n* = 3) or isolated T cells (*n* = 6). The scatter dot plot represents the effect of small molecules on cellular viability calculated using the AUC model of log transformed dose-responses data using GraphPad V7. The line in the box-and-whisker diagram represents the AUC median. The upper edge (hinge) of the box indicates the 75th percentile of the data, and the lower hinge the 25th percentile. The ends of the vertical line indicate the minimum and the maximum data values. Statistical significance (*P ≤ 0.05) was determined by a non-parametric *t*-test (Mann–Whitney). **J** Effect of G9a inhibitors BIX01294, UNC0638, and UNC0642 in CD25^+^ thymic cells isolated from CD1 mice or PDLX-0122 T-ALL cells. Cells were grown at increasing concentrations of G9a/GLP inhibitors (BIX01294, UNC0638, and UNC0642) and viability evaluated at day 3 by an ATP-based assay and plotted as the percentage of viable cells. Shown is the mean ± standard deviation (SD) of a minimum of three replicates.

To identify candidate therapeutic targets in T-ALL, we evaluated inhibitors of epigenetic modifiers, including modulators of the histone acetyltransferase (HAT) p300 (CTPB and C646) and inhibitors of protein methyltransferases (PMTs), small molecules targeting EZH2 (GSK126), DOT1L (SGC0946), and G9a (*EHMT2*)/G9a-like-(GLP) (*EHMT1*) (UNC0638 and BIX01294). G9a/GLP inhibitors significantly impaired cell viability compared to the other molecules screened, as measured by the reduction of cellular ATP content, and quantified by a lower area under the curve (AUC) (Fig. 1A and Supplementary Fig. 1A–C). We next determined whether the effect of G9a inhibition, including with UNC0642, a second-generation derivative of UNC0638 optimized for high selectivity and in vivo potency against G9a and GLP [6], is more profound in the lymphoblastic lineage compared to other leukemias (e.g., myeloid) or cancer types. Indeed, analysis of the Genomics of Drug Sensitivity in Cancer database [14] or drug testing in clinical leukemia samples or cell lines revealed a preferential activity of G9a inhibitors in T-ALL compared to other tumor types (Fig. 1B, C and Supplementary Fig. 1D, E) (IC50, BIX01294 mean = 2.52 ± 1.81 μM, 0.63 < *x* < 6.88; UNC0638 mean = 2.53 ± 1.16 μM, 1.18 < *x* < 5.03; UNC0642 mean = 3.49 ± 1.54 μM, 1.36 < *x* < 6.04).

To compare expression patterns of *EHMT2* in T-ALL to other cancers and normal cellular states, we analyzed several transcriptional databases, including the Differentiation Map (DMAP) [15], Cancer Cell Line Encyclopedia (CCLE) [16], and primary T-ALL datasets [17, 18]. DMAP analysis showed a comparatively higher expression of *EHMT2* during CD4^+^/CD8^+^ T-cell lineage hematopoietic commitment (Supplementary Fig. 1F) and supports the hypothesis of a preferential cancer dependency based on hematopoietic lineage commitment. T-ALL displayed higher *EHMT2*/G9a expression compared to other tumor types [16] (Fig. 1D), normal human thymocytes (Fig. 1E, Supplementary Fig. 1G), or lymphocytes (Fig. 1F) independent from the activation of known transcription factor or recurrent mutations (Supplementary Fig. 1H). G9a is expressed in T-ALL cell lines, including the two spliced isoforms, long (G9a-l) and short (G9a-s) [10] (Fig. 1G). Consistent with the differential distribution of G9a in normal and lymphoblast cells, T-ALL were significantly more sensitive compared to lymphocytes (Fig. 1H, I) or to CD25^+^ thymocytes isolated from CD1 mice (Fig. 1J).

### G9a blockade alters T-ALL proliferation, in vitro, 3D, and preclinical T-ALL models

**Fig. 2:**
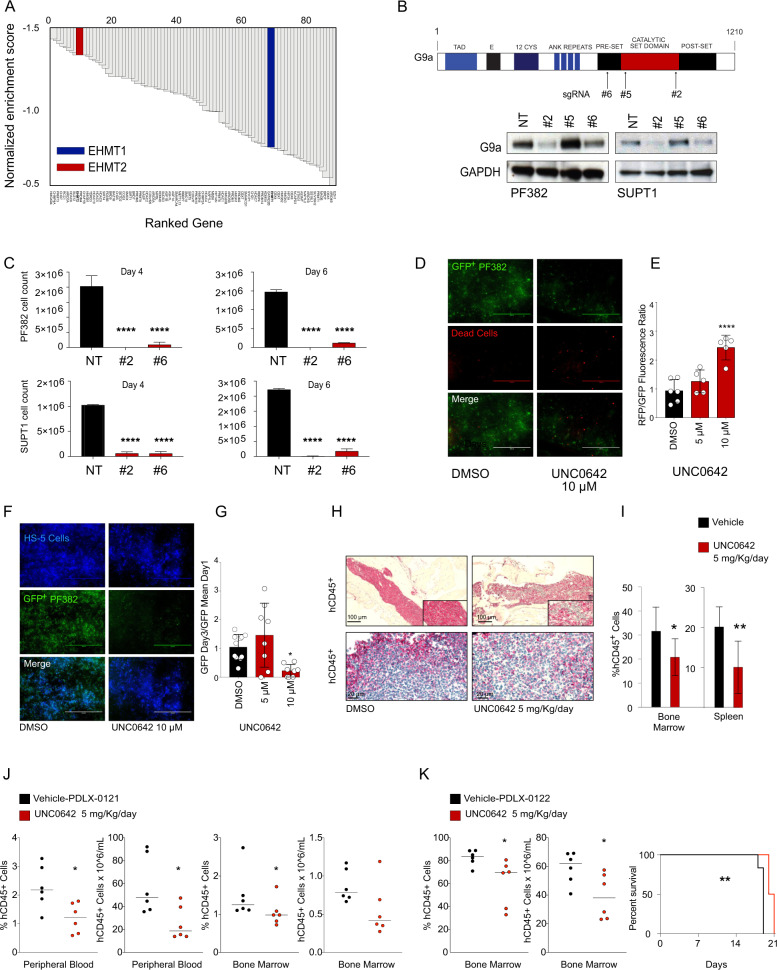
G9a enzymatic abrogation impairs T-ALL proliferation in vitro, 3D and in vivo preclinical T-ALL models. **A** shRNA screen identified that *EHMT2* is required for T-ALL cell survival. Top hits are ranked with a negative Normalized Enrichment Score (NES). shRNA screen was performed in DND41 T-ALL cell line [19]. **B** Schematic representation of the G9a protein with the indicated functional domains (top). On the bottom, western blot showing expression of G9a in PF382 and SUPT1 cells 2 days post sgRNA selection. Protein lysates were stained with an anti-G9a antibody. GAPDH was used as loading control. NT = non-targeting. **C** Effect of G9a loss in PF382 and SUPT1 cells at four or six days post sgRNA selection. Histograms show cell count using the trypan blue exclusion assay. Error bar denotes the mean ± SD of a minimum of three biological replicates. Statistical significance among groups (****P ≤ 0.0001) was determined by a non-parametric *t*-test (Mann–Whitney). NT = non-targeting, #2 = sgRNA #2 directed against *EHMT2*. **D** Live Dead assay of GFP + PF382 in 3D cell culture treated with DMSO or UNC0642 at the indicated concentrations. Representative immunofluorescence images of control or UNC0642 treated PF382 cells upon Live/Dead® staining at 72 h. Scale bars: 1000 μm. **E** Cell viability assay of GFP + PF382 cell in 3D culture treated with DMSO or UNC0642 at the indicated concentrations. Cell death is indicated in the histogram as a fluorescence ratio between GFP + (viable cells) and RFP + (dead cells) signals of the acquired fields. Error bars denote the mean ± standard deviation (SD) of one representative experiment. Statistical significance among groups for treated vs. vehicle (DMSO) (****P ≤ 0.0001) was determined by one-way ANOVA using Bonferroni’s correction for multiple comparison testing. **F** Preclinical validation of UNC0642 in PF382 co-cultured with HS-5 human stromal cells in 3D scaffolds. Representative immunofluorescence images of control or UNC0642 treated PF382 (green) cultured in 3D scaffolds with stromal HS-5 cells (blue) at Day 3. Scale bars: 1000 μm. **G** Preclinical validation of UNC0642 in PF382 co-cultured with HS-5 human stromal cells in 3D scaffolds. The histogram shows the effect of UNC0642 treatment on PF382 cell viability as represented by a GFP fold increase relative to day 1. Error bars denote the mean ± standard deviation (SD) of one representative experiment. Statistical significance among groups for treated vs. vehicle (DMSO) (*P ≤ 0.05) was determined by one-way ANOVA using Bonferroni’s correction for multiple comparison testing. **H** Antileukemic activity of UNC0642 in hCD45+ bone marrow infiltrating cells (top panels) and spleen (bottom panel) in a MOLT16 xenografted murine model after 12 days of UNC0642 treatment (5 mg/kg/IP every 48 h) or vehicle (corn oil). Formalin-fixed, paraffin embedded tissue sections were stained with anti hCD45 antibody. Scale bars, 100 or 20 μm. **I** The number of hCD45+ cells per field was represented as percentage relative to vehicle control. Error bars denote the mean ± SD of eight fields from three representative mice treated with UNC0642 or the mean ± SD of three fields from three representative vehicle treated mice. Statistical significance for treated vs. vehicle (*P ≤ 0.05, **P ≤ 0.01) was determined by non-parametric *t*-test (Mann–Whitney). **J** Antileukemic activity of UNC0642 in hCD45 + T-ALL leukemia cells (bone-marrow and peripheral cells T-ALL lymphoblasts) in a PDLX T-ALL, #0121, murine model after 26 days of UNC0642 treatment (5 mg/kg/IP every 48 h) or vehicle (corn oil). Statistical significance for treated vs. vehicle (*P ≤ 0.05) was determined by non-parametric *t*-test (Mann–Whitney). **K** Antileukemic activity of UNC0642 in hCD45 + T-ALL leukemia cells (bone-marrow and peripheral cells T-ALL lymphoblasts) in a PDLX T-ALL, #0122, murine model after 9 days of UNC0642 treatment (5 mg/kg/IP every 48 h) or vehicle (corn oil). Statistical significance for treated vs. vehicle (*P ≤ 0.05) was determined by non-parametric *t*-test (Mann–Whitney). On the right Kaplan–Meier survival plots showing the overall survival (OS) of PDLX T-ALL mice treated or untreated with UNC0642 (5 mg/kg/IP every 48 h) (**P ≤ 0.01).

To validate G9a as a therapeutic target in T-ALL, we intersected our results with a previously published short hairpin RNA (shRNA) screen targeting nearly 350 chromatin regulator genes in DND41 T-ALL cell line [19]. *EHMT2* scored among the top 10 hits that impaired the viability of T-ALL cells (Fig. 2A), indicating that T-ALL relies on *EHMT2* expression. Because shRNA-based screens are limited by the occurrence of off-target effects, we took a CRISPR approach for target validation in two validated Cas9 expressing models (PF382 and SUPT1). sgRNAs targeting the enzymatic SET domain of G9a diminished G9a expression (Fig. 2B) and resulted in decreased cell viability as measured by trypan blue exclusion (Fig. 2C) and an ATP-based assay (Supplementary Fig. 2A) compared to a non-active sgRNA guide (#5) (Supplementary Fig. 2B). Loss of G9a induced apoptotic cell death measured by Annexin V/propidium iodide (PI) staining (Supplementary Fig. 2C).

Next, we created a 3D leukemia model (Supplementary Fig. 2D) in a bioreactor device, (VITVO) [20] to recapitulate the complexity of the bone marrow microenvironment, which can impair response to small-molecule therapies. Cells were treated for 72 h with UNC0642, at 5 and 10 μM. UNC0642 treatment caused a dose-dependent decrease in T-ALL viability measured by an ATP-based assay and GFP imaging quantification (Fig. 2D, E, Supplementary Fig. 2E–G). We also seeded mesenchymal stromal cells (HS-5) in the inner layer of the 3D VITVO chamber and 24 h later we loaded PF382-GFP positive cells. HS-5 cells easily attached and colonized the scaffold matrix promoting the formation of visible three-dimensional architecture with PF382 cells after 24 h of co-culture (Fig. 2F). UNC0642 treatment caused a viability defect even in the context of a leukemia-stroma interaction (Fig. 2F, G), consistent with the observation that G9a inhibitors force T-ALL to undergo apoptosis (Supplementary Fig. 2H).

Next, we established an orthotopic xenograft from a T-ALL cell line. After disease establishment, mice were treated for 12 days at a dose of 5 mg/kg every other day with UNC0642 versus a vehicle control by intraperitoneal injection. UNC0642 treatment resulted in a reduction of the percentage of hCD45 positive MOLT16 cells in the bone marrow of xenotransplanted mice compared to the vehicle-administered control group and, consistently, a reduction of leukemic infiltration in the spleen (Fig. 2H, I). These studies prompted further in vivo experiments where we demonstrated that UNC0642 reduces leukemic burden both in a slowly growing (Fig. 2J) or fast growing (Fig. 2K) T-ALL patient-derived leukemia xenograft (PDLX) models.

These data credential *EHMT2* as a candidate therapeutic target in T-ALL and demonstrate that UNC0642 reduces T-ALL growth in xenograft models supporting further preclinical optimization of G9a inhibitors and evaluation in additional T-ALL models.

### G9a inhibition modulates H3K9me1-2 in T-ALL

G9a primarily exerts its transcriptional silencing by targeting the mono and dimethylation of lysine 9 at histone-3 (H3K9), while methylation on H3 residues different from lysine 9 (e.g., H3K27) has been rarely described [7].

**Fig. 3:**
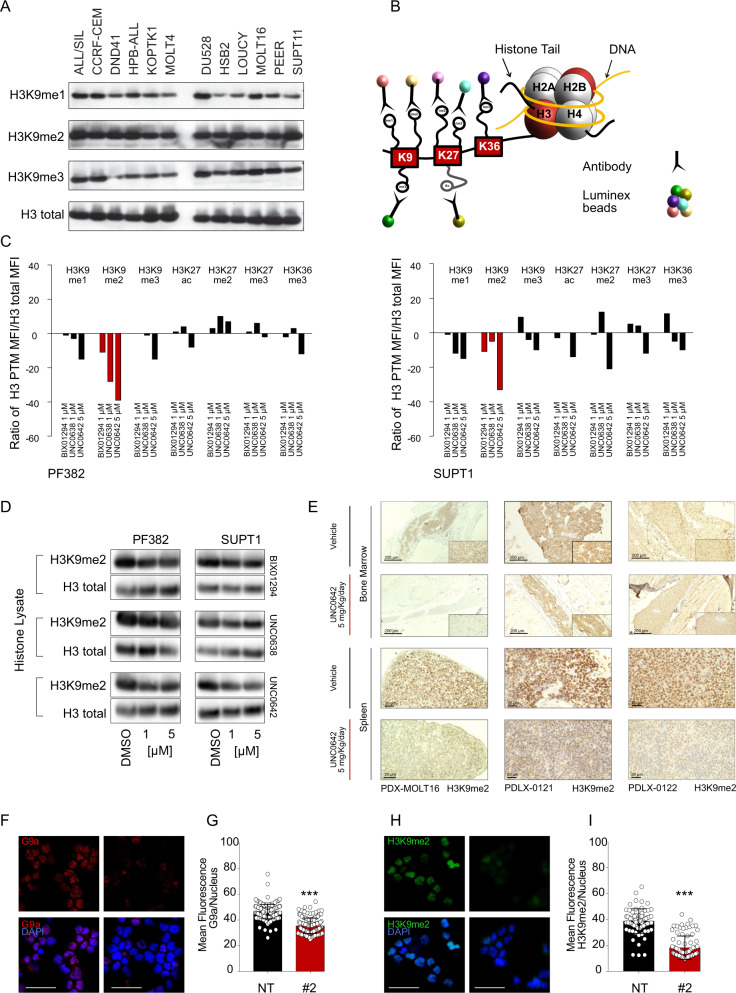
G9a inhibitors reduce H3K9me2 level in T-ALL. **A** Western blot showing expression of H3K9me1, H3K9me2, and H3K9me3 in a panel of T-ALL cell lines. An acidic histone protein extraction was performed. Total histone-3 (H3) was used as a loading control. **B** Schematic representation of the histone protein methyltransferase, Luminex beads assay. The beads/target panel included H3K9me1, H3K9me2, H3K9me3, H3K27ac, H3K27me2, H3K27me3, and H3K36me3. **C** Luminex PMT assay. Lysates were obtained from acidic extraction upon 48 h treatment with the indicated concentrations of G9a inhibitors. Histograms show the median fluorescence intensity of each histone mark normalized to the intensity of total H3. Detection was repeated three times by diluting initial protein concentration with a 2.5-dilution series. Graphs are representative of one out of three Luminex quantifications. Red bar indicates H3K9me2 quantification values. **D** Western blot showing expression of H3K9me2 in PF382 and SUPT1 cells treated at the indicated concentrations of G9a inhibitors for 48 h. Protein lysates were obtained using an acidic histone extraction protocol and stained with an antibody recognizing the H3K9me2 residue or total H3 used as a loading control. **E** Modulation of H3K9me2 in xenografted murine models after 12 (PDX-MOLT16), 26 (PDLX-0121), 9 (PDLX-0122) days of UNC0642 treatment (5 mg/kg/IP every 48 h) or vehicle (corn oil). Top panel = bone marrow, bottom panel = spleen. Scale bar: 20 μm. Formalin-fixed, paraffin embedded tissue sections were stained with anti H3K9me2. Scale bars, 200 or 20 μm. **F** Effect of *EHMT2* knock down on H3K9me2 in PF382 cells 2 days post sgRNA selection. Immunofluorescence of permeabilized PF382 cells stained with anti-G9a (red) and anti-H3K9me2 (green) (H) is shown. Cell nuclei were stained with DAPI (blue). Scale bar: 100 μm. NT = non-targeting, #2 = sgRNA #2 directed against *EHMT2*. **G** Quantitative immunofluorescence analysis of G9a/*EHMT2* and H3K9me2. **I** Nuclear signal in PF382 cells after 2 days post sgRNA selection. Error bars denote the mean ± standard deviation (SD) of fluorescence of single nucleus (arbitrary units); Statistical significance among NT vs. sgRNA #2 was determined by unpaired *t*-test (***P ≤ 0.001).

To ensure that these drugs efficiently inhibit the histone methyltransferase activity of G9a in T-ALL, we first quantified the H3K9 methyl marks (me1-3) upon acid histone extraction and found homogenous levels of methylated residues in both *NOTCH1* mutated and wild-type T-ALL cell lines (Fig. 3A). Next, we performed a bead-based histone post-translational modification assay to simultaneously measure multiple histone modification targets (Fig. 3B). In both PF382 and SUPT1 cell lines we observed a decrease in H3K9me2 with each of the G9a inhibitors (Fig. 3C). Consistent with previous reports, UNC0642 was the most robust G9a inhibitor [9]. We then analyzed acid-extracted nuclear histone fractionated proteins and whole-cell lysates derived from six T-ALL cell lines treated with increasing concentrations of G9a inhibitors, DMSO or the Notch Inhibitor Compound E as a negative control by western blot, confirming a decrement of H3K9me2 (Fig. 3D, Supplementary Fig. 3A–F). Furthermore, UNC0642 treatment in vivo modulated the H3K9me2 mark in the bone marrow and spleen of xenotransplanted mice compared to the vehicle-administered control group (Fig. 3E). Consistently, sgRNA guided genetic loss of *EHMT2* caused a decrement of H3K9me2 levels (Fig. 3F–I). These data support the on-target activity of both the small molecules and genetic perturbation of *EHMT2* as assessed by altered levels of H3K9me2.

### G9a loss suppresses Sestrin2 in T-ALL

**Fig. 4:**
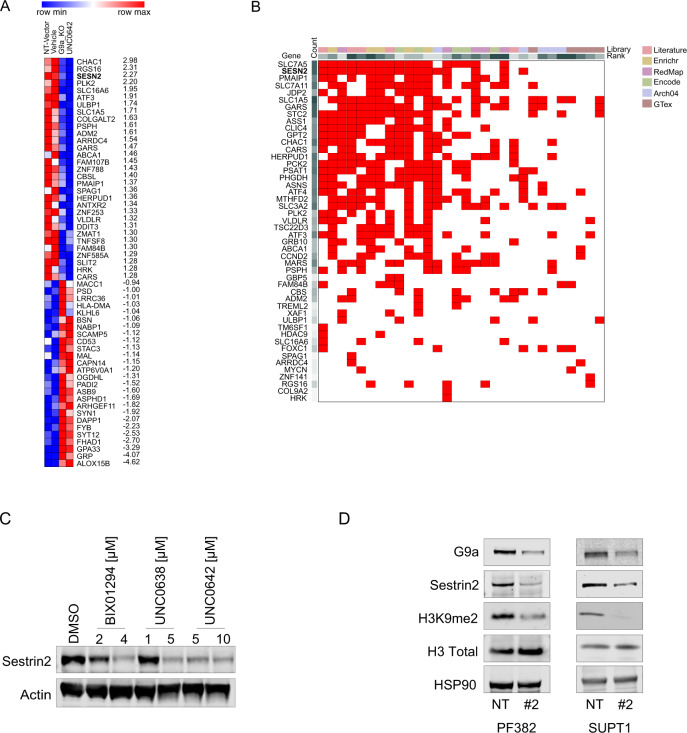
Transcriptional consequences of G9a loss in T-ALL. **A** Heatmap showing mRNA expression of differentially expressed genes in non-targeting sgRNA (sgNT), sgRNA targeting *EHMT2* (#2) or T-ALL cells treated either with vehicle or UNC0642 (*P* < 0.05 and Log_2_ fold changes > 1.5) and ranked for signal to noise ratio (snr). Each column represents the mean of two biological replicates per condition. **B** Clustergram showing transcription factor enrichment analysis (TFEA). G9a loss gene set from (**A**) was compared to the ChEA3 benchmark libraries as listed in https://amp.pharm.mssm.edu/chea3/#top for TF target over-representation analysis. Red boxes show the overlapping query gene targets among top library results. **C** Western blot showing expression of sestrin2 in PF382 cells treated at the indicated concentrations of G9a inhibitors after 48 h. Actin was used as a loading control. **D** Western blot showing expression of G9a/*EHMT2*, sestrin2, H3K9me2, and H3 Total, in PF382 and SUPT1 cells after 2 days post sgRNA selection. HSP90 was used as a loading control. NT non targeting; #2 = sgRNA #2 directed against *EHMT2*.

To identify pathways associated with suppression of G9a, we investigated the transcriptional consequence of G9a loss in RNA isolated from PF382 cells, expressing either non-targeting control sgRNA (sgNT), sgRNA #2 or treated with either vehicle or UNC0642. We performed a regression analysis on the significant differentially expressed genes, which showed high correlation between the *EHMT2* knock down and UNC0642 treatment (*R*^2^ = 0.78, Supplementary Fig. 4A). We established a G9a signature of mRNA transcripts repressed in sgRNA #2 or UNC0642 treatment and ranked the top 50 by the signal to noise ratio (snr) (Fig. 4A)_._ G9a modulation significantly (*P* < 0.05; Log_2_ fold-change ≤ −0.5 or ≥ 0.5) altered expression of 3% of transcribed genes (1448); with nearly 55.5% downregulated and 44.5% upregulated, supporting the notion that, depending on tumor types [21, 22], G9a mediated H3K9 methylation can both promote and suppress gene transcription. Next, we compared our G9a signature with previously reported G9a/upregulated/downregulated gene sets (GSE113493, GSE118992, GSE51512, GSE34925, GSE70914, GSE41226) using gene set enrichment analysis (GSEA) [23]. G9a transcriptional changes were significantly enriched in G9a-modulated gene sets (*P* < 0.01, false discovery rate < 0.05) (Supplementary Fig. 4B), suggesting a set of genes similarly regulated by G9a across different cancer types. To identify genes that contributed most strongly to the *EHMT2* gene sets, we applied leading edge analysis [23] to the G9a signatures (Supplementary Fig. 4C) and transcription factor (TF) enrichment analysis (TFEA), a method (Fig. 4B) that prioritizes transcription factors based on the overlap between a given list of differentially expressed genes, and previously annotated TF targets assembled from ChIP-Seq experiments, RNA studies performed in different tissues and conditions [24]. Both algorithms identified sestrin2 (*SESN2*) as a potential target of *EHMT2*. Interestingly, *SESN2* was identified in gene ontology (GO) pathways linked to cellular stress, such as the “PERK-mediated unfolded protein response” (GO:0036499; 6 counts; adj. *P* = 8.868987e-05), “response to endoplasmic reticulum stress” (GO:0034976; 16 counts; adj. *P* = 8.868987e-05), “intrinsic apoptotic signaling pathway in response to endoplasmic reticulum stress” (GO:0070059; eight counts; adj. *P* = 2.588436e-04), “cellular response to starvation” (GO:0009267; 10 counts; adj. *P* = 1.161513e-03) (Supplementary Fig. 4D). These results suggest that G9a loss may sensitize cells to the activation of cell death and stress/autophagic related pathways.

To validate our transcriptional data, we repressed G9a catalytic activity (Fig. 4C and Supplementary Fig. 4E), or expression (Fig. 4D), and demonstrated that *EHMT2*/G9a inactivation caused a decrement in sestrin2 expression in multiple T-ALL cell lines. Because sestrin2 is a stress-induced protein involved in different cellular adaptive responses by regulating ER stress, inflammation, and autophagy [25], we decided to explore the contribution of *SESN2* repression to the phenotypic changes caused by G9a suppression.

### G9a modulates glycolysis rate in T-ALL

Several studies suggest that sestrin2 inhibition leads to a significant decrease of ATP cellular levels [26, 27] and aggravates stress induced death pathways associated with glucose starvation and the inhibition of glycolysis [27]. First, we genetically suppressed *SESN2* (Supplementary Fig. 5A) and demonstrated, T-ALL cells undergo apoptosis (Supplementary Fig. 5B) similarly to T-ALL cells lacking G9a (Supplementary Fig. 2C).

Next, we investigated the consequences of G9a/sestrin2 axis inhibition on glycolysis using the Agilent Seahorse XFp assay. In this assay, the glycolytic rate is evaluated by measuring, in real time, the Extracellular Acidification Rate (ECAR). ECAR correlates with the conversion of glucose to lactate, which results in the net production of protons that exit the cells. Because mitochondrial respiration contributes to the acidification of the cell medium by producing CO_2_ (which is partially hydrated in the extracellular medium), the Proton Efflux Rate (PER, a measurement of the extracellular acidification rate) was obtained both under basal condition and following inhibition of the mitochondrial function by rotenone and antimycin A (Rot/AA). The contribution of mitochondria/CO_2_ to extracellular acidification was subtracted from PER to obtain the glycoPER which is the rate of protons extruded in the extracellular medium during glycolysis (Supplementary Fig. 5C).

**Fig. 5:**
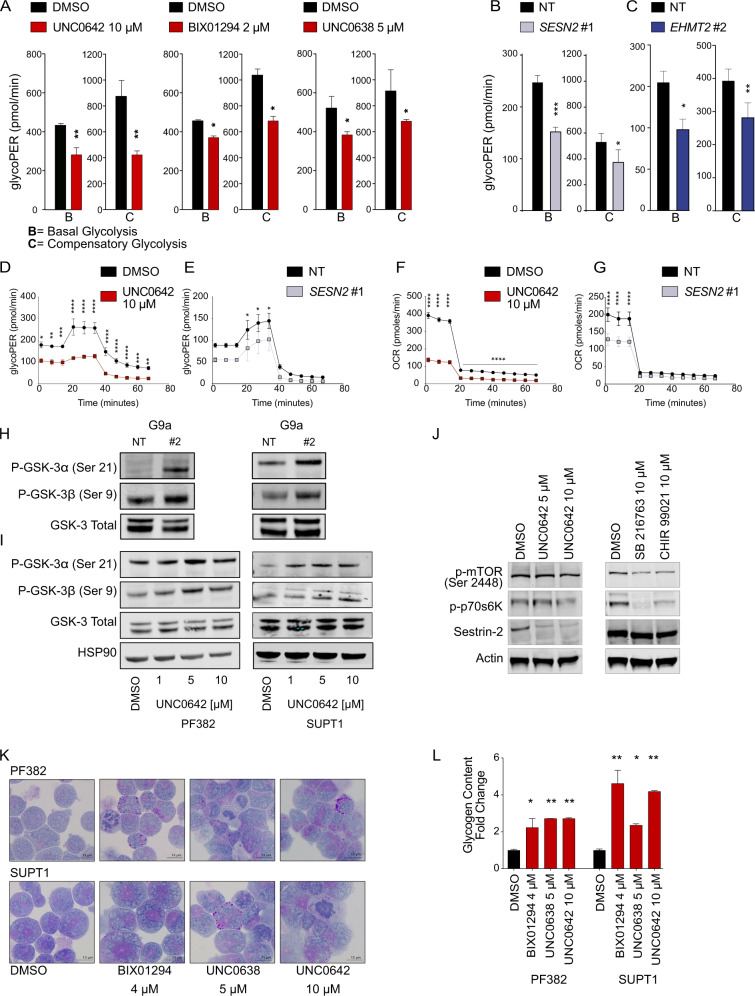
G9a/*EHMT2* modulates Glycogen Synthase Kinase-3 (GSK-3) in T-ALL. **A** Glycolytic phenotype in PF382 cells. Histograms represent mean ± SD of three replicates of PF382 T-ALL cells treated with DMSO, BIX01294 (2 μM), or UNC0638 (5 μM) or UNC0642 (10 μM) on the x-axis. The y-axis represents the glycolytic proton efflux rate (PER) at the basal and compensatory level. Statistical significance among groups (*P < 0.05, **P < 0.01) was determined by a non-parametric *t*-test (Mann–Whitney). **B** Glycolytic phenotype in *SESN2* knock down PF382 cells. Histograms represent mean ± SD of three replicates of *SESN2* deprived T-ALL cells. The y-axis represents the glycolytic proton efflux rate (PER) at the basal and compensatory level. Statistical significance among groups (*P < 0.05, ***P < 0.001) was determined by a non-parametric *t*-test (Mann–Whitney). **C** Glycolytic phenotype in *EHMT2* knock down PF382 cells. Histograms represent mean ± SD of three replicates of *EHMT2* deprived T-ALL cells. The y-axis represents the glycolytic proton efflux rate (PER) at the basal and compensatory level. Statistical significance among groups (*P < 0.05, **P < 0.01) was determined by a non-parametric *t*-test (Mann–Whitney). **D** Time resolved glycolytic proton efflux rate (glycoPER). Traces represent the mean ± SD of three replicates of PF382 cells treated with DMSO or UNC0642 analyzed over 70-min time course experiments. Statistical significance among groups for treated vs. vehicle (DMSO) (*P < 0.05, **P < 0.01, ***P < 0.001, ****P < 0.0001) was determined by two-way ANOVA using Bonferroni’s correction for multiple comparison testing. **E** Time resolved glycolytic proton efflux rate (glycoPER). Traces represent the mean ± SD of three replicates of *SESN2* knock down PF382 analyzed over 70-min time course experiments. Statistical significance among groups for treated vs. vehicle (DMSO) (*P < 0.05) was determined by two-way ANOVA using Bonferroni’s correction for multiple comparison testing. **F** Oxygen Consumption Rate (OCR). Traces represent the mean ± SD of three replicates of PF382 T-ALL cells treated with DMSO or UNC0642 analyzed over a 70-min time course experiment. Statistical significance among groups for treated vs. vehicle (DMSO) (****P < 0.0001) was determined by two-way ANOVA using Bonferroni’s correction for multiple comparison testing. **G** Oxygen Consumption Rate (OCR). Traces represent the mean ± SD of three replicates of PF382 *SESN2* knockdown cells analyzed over a 70-min time course experiment. Statistical significance among groups for treated vs. vehicle (DMSO) (****P < 0.0001) was determined by two-way ANOVA using Bonferroni’s correction for multiple comparison testing. **H** Modulation of GSK-3 in PF382 and SUPT1 cells after 2 days post sgRNA selection. Western blot showing expression phosphorylated serine-9 in GSK-3β or serine-21 in GSK-3α in T-ALL. Protein lysates were stained with G9a/*EHMT2*, P-GSK-3α (Ser21), P-GSK-3β (Ser9), GSK-3 total. NT non targeting, #2 = sgRNA #2 directed against *EHMT2*. See Fig. 6E for *EHMT2* knock down level. **I** Modulation of GSK-3 by the G9a inhibitor UNC0642. Western blot showing phosphorylation of serine-9 in GSK-3β or serine-21 in GSK-3α in T-ALL. Cell lysates were obtained after 48 h of drug treatment as indicated. Protein lysates were stained with P-GSK-3α (Ser21), P-GSK-3β (Ser9), and GSK-3 Total. HSP90 was used as loading control. **J** Western blot showing level of p-mTOR (Ser-2448), sestrin2, and p-p70s6K in PF382 cells after UNC0642 treatment or 24 h of GSK-3 inhibitor (SB216763 and CHIR-99021) treatment at the indicated concentration. Actin was used as a loading control. **K** Micrographs documenting Periodic Acid Shift (PAS) staining in PF382 and SUPT1 cell lines treated with vehicle (DMSO) or the indicated doses of BIX01294, UNC0638, and UNC0642 for 48 h. Fine dispersed granular or blocks of PAS + (magenta) cytoplasmic and perinuclear material correspond to glycogen. Scale bars: 13 µm. **L** Effect of G9a inhibitors BIX01294, UNC0638, and UNC0642 on cellular glycogen content. Histograms show the glycogen fold change increase relative to a DMSO control after G9a inhibitors treatment for 48 h. Error bars denote the mean ± standard deviation (SD) of two biological replicates. Statistical significance among groups (*P ≤ 0.05, **P ≤ 0.01) was determined by a non-parametric *t*-test (Mann–Whitney).

Basal and compensatory glycolysis rates (i.e., glycolysis rate measured following mitochondrial inhibition) were higher in vehicle versus UNC0642, UNC0638, and BIX01294 treated cells (Fig. 5A, Supplementary Fig. 5D), *SESN2* (Fig. 5B) and *EHMT2* knockdown cells (Fig. 5C, Supplementary Fig. 5E) suggesting that G9a inhibition decreases the glycolytic flux in T-ALL.

We observed a trend toward a lower glycoPER in UNC0642 treated (Fig. 5D), *SESN2* (Fig. 5E), and *EHMT2* (Supplementary Fig. 5F and 5H) deprived T-ALL cells versus DMSO or non-targeting control, confirming a decrease in overall glycolysis following G9a inhibition. Consistent with this, vehicle controls had higher oxygen consumption rate (OCR) compared to UNC0642 treated (Fig. 5F), *SESN2* (Fig. 5G) and *EHMT2* (Supplementary Fig. 5G and 5I) knockdown T-ALL cells indicating that G9a inhibition may also negatively affect the rate of mitochondrial respiration and therefore the capability of the cells to compensate for the inhibition of glycolysis with an increase of the oxidative phosphorylation. Collectively these data suggest a role of G9a in the control of cellular energy metabolism at least in part mediated by sestrin2 as demonstrated in the gene signature analysis and western blot assays following G9a suppression.

A consequence of defective glycolysis and ATP depletion is the feedback activation of the AKT pathway energy sensors AMP-activated protein kinase (AMPK) [28] and, ultimately, the inactivation of the glycogen synthase kinase-3 (*GSK-3A/B*) [29]. GSK-3 regulates the activity of Glycogen Synthase (GS), an enzyme that mediates the conversion of glucose to glycogen. GSK-3 consists of two serine-threonine kinase α and β paralogues and is involved in several cellular processes [30**–**32]. GSK-3 is constitutively active in resting cells; phosphorylation at GSK-3β/α Y216/279 is activating and phosphorylation at GSK-3β/α S9/21 is inhibitory. Lysates from G9a knockdown cells showed an increase in phosphorylation in GSK-3β/α S9/21 (Fig. 5H) inhibitory sites with a preferential effect on GSK-3α (Supplementary Fig. 6A). Similarly, lysates from G9a inhibitor treated cells revealed similar changes in the phosphorylation levels of the GSK-3β/α S9/21 sites, consistent with the inhibition of GSK-3 kinase activity (Fig. 5I and Supplementary Fig. 6B). These events are independent from the inhibition of the mTOR axis, yet consistent with previous reports [27], as shown by the comparative analysis of the effects of G9a and GSK-3 inhibitors (Fig. 5J) and the lack of modulation on p-AKT and p-AMPK (Supplementary Fig. 6C).

Because GSK-3 inhibition has been previously shown to increase cellular glycogen content [32], we asked whether G9a inhibitor treated cells displayed glycogen accumulation in T-ALL. G9a inhibition leads to an accumulation of Periodic Acid-Schiff (PAS) positive complexes (Fig. 5K), glycogen-rich electron-dense bodies in autophagosomes (Ap) (Supplementary Fig. 6D), and results in the net accumulation of glycogen (Fig. 5L and Supplementary Fig. 6E, F).

These data suggest that G9a loss is critical to the survival of T-ALL by controlling cellular energy metabolism.

### G9a suppression triggers autophagy in T-ALL

G9a modulation has been reported to induce a variety of phenotypic changes in a cell context dependent manner [12] and, among the most consistent, G9a inhibition promotes autophagy-associated apoptosis [21, 33]. Because glycogen accumulation leads to altered autophagosomes and lysosomes fusion or degradation [34], we asked whether the metabolic consequences of G9a suppression may alter autophagy in T-ALL.

**Fig. 6:**
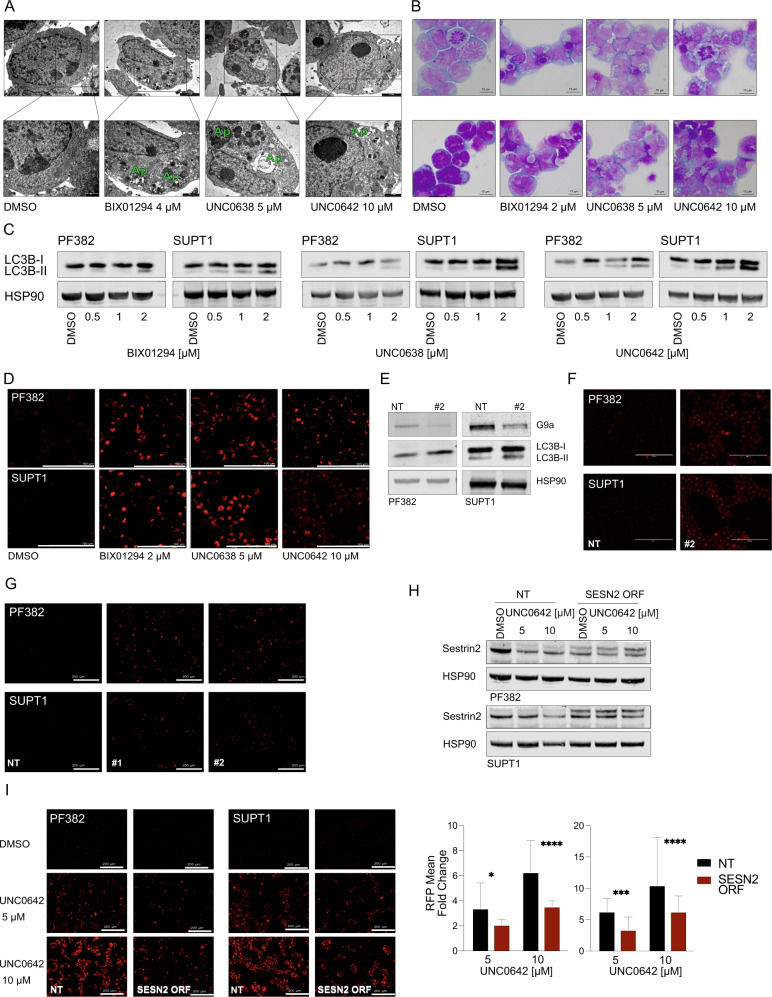
G9a suppression triggers apoptosis and autophagy in T-ALL. **A** TEM micrographs illustrating the morphology of untreated (DMSO) T-ALL cells and the effects of BIX01294, UNC0638, and UNC0642. Black rectangles (upper) inscribe areas shown at higher magnification (lower) to illustrate the phases of autophagosome (Ap) formation with G9a inhibitions. Scale bars: upper panels DMSO 1 µm, G9a inhibitors 2 µm; lower panels DMSO 0.5 µm, G9a inhibitors 1 µm. **B** Representative images of May–Grunwald–Giemsa stained cytospin preparations of T-ALL (PF382 and SUPT1) cultured in vehicle (DMSO) or BIX01294, UNC0638, or UNC0642 for 48 h. Extensive cytoplasmic vacuolization is apparent following exposure to G9a inhibitors. Images were captured with a Leica ICC50W optical microscope (100X). Scale bars: 13 µm. **C** Western blot showing expression of LC3B-II in PF382 and SUPT1 cells treated at the indicated concentrations of G9a inhibitors for 48 h. HSP90 was used as a loading control. **D** LysoTracker® Red DND-99 staining of T-ALL cells labeled treated with BIX01294, UNC0638, and UNC0642 treatment for 48 h. Scale bars: 100 µm. **E** Western blot showing expression of G9a, 2 days post sgRNA selection. Protein lysates were blotted with a G9a, LC3B-II antibody, HSP90 was used as loading control. NT non-targeting, #2 = sgRNA #2 directed against *EHMT2*. **F** LysoTracker® Red DND-99 staining of T-ALL cells 2 days post sgRNA selection. Scale bar: 100 μm. NT non-targeting, #2 = sgRNA #2 directed against *EHMT2*. Scale bars: 100 µm. **G** LysoTracker® Red DND-99 staining of T-ALL cells 2 days post selection for shRNA targeting *SESN2* selection. Scale bar: 200 μm. **H** Western blot showing expression of sestrin2 in wild type or cDNA-*SESN2*-orf PF382 and SUPT1 cells treated at the indicated concentrations of G9a inhibitors for 48 h. HSP90 was used as a loading control. **I** LysoTracker® Red DND-99 staining in wild type or cDNA-*SESN2*-orf PF382 and SUPT1 cells treated at the indicated concentrations of G9a inhibitors for 48 h. Scale bars: 200 µm (left). Histograms show the mean and standard deviation of RFP per field (*n* = 10) relative to the vehicle control. Statistical significance among groups (*P < 0.05, ***P < 0.001, ****P < 0.0001) was determined by a non-parametric *t*-test (Mann–Whitney).

Transmission electron microscopy (TEM) analysis showed an accumulation of autophagic vesicles in apoptotic PF382 cells (Fig. 6A, Supplementary Fig. 7A), with otherwise intact organelles (Fig. 6A). Consistent with this finding May–Grunwald–Giemsa (MGG) stained PF382 and SUPT1 cells treated with G9a inhibitors for 48 h showed an increased accumulation of vacuoles in the cytoplasm relative to DMSO control treatment (Fig. 6B). To biochemically validate our ultra-structural analysis, we demonstrated that all three G9a inhibitors cause a dose-dependent increase in the microtubule-associated light chain 3 (LC3) form (II) protein levels bound to autophagosomes at 48 h post-treatment (Fig. 6C). These effects were not seen in T-ALL treated with chemotherapy or targeted agents (Supplementary Fig. 7B, C).

In addition to autophagosomes, identified by the presence of a double membrane, TEM analysis revealed an increased number of lysosomes (Supplementary Fig. 7D).

To provide further evidence of induction of autophagy by G9a inhibitors in T-ALL cells, we performed acidotropic staining with LysoTracker Red dye and observed a significant increase in the number of acidic vesicles, indicative of autophagosome/lysosome fusion in cell lines (Fig. 6D), a process reverted with the addition of Bafilomycin A1 a known inhibitor of autophagosomes and lysosome fusion (Supplementary Fig. 8A). Similarly, G9a inhibition triggered autophagy in cells growing in 3D matrices (Supplementary Fig. 8B–E). Furthermore, genetic suppression of G9a levels (Fig. 6E) caused the formation of autophagic acidotropic vesicles in T-ALL cell lines (Fig. 6F). Because glycogen accumulation may result in disruptive autophagosomes and lysosomes formation/degradation cycle [35] we repeated the acidotropic staining in the presence or absence of 2-Deoxy-d-glucose (2-DG) to lock the activity of hexokinase and inhibit the access of glucose for glycolysis and glycogen formation. As shown in Supplementary Fig. 8F the addition of 2-DG rescues the autophagosome formation suggesting that the accumulation of glycogen is partially responsible for the observed phenotype in G9a treated cells.

The next question was to determine whether sestrin2 repression plays a role in the autophagy observed with G9a loss. We therefore in genetically suppressed *SESN2* and demonstrated that in *SESN2* deficient cells (Supplementary Fig. 5A) the lysotracker probe accumulates in acidotropic vesicles (Fig. 6G), similarly to T-ALL cells lacking G9a. Moreover, forced expression of cDNA-*SESN2-*orf in PF382 and SUPT1 cells (Fig. 6H) partially rescued T-ALL cells from UNC0642 mediated effect on autophagosomes formation (Fig. 6I).

## Discussion

Following the recent success of inhibitors of DNA methyltransferases (DNMTs) and histone deacetylases (HDACs), some of which are now clinically approved [36], a new wave of epigenetic targeting molecules focused on histone methyltransferase inhibitors have entered clinical investigation. DOT1L, EZH2, and LSD1 inhibitors are currently being evaluated in clinical trials with evidence of therapeutic activity [37], underscoring their promise as anti-cancer agents.

We credential *EHMT2*/G9a, a SET domain-containing histone methyltransferase, as a potential therapeutic target in T-ALL and focus on elucidating and linking the role of G9a inhibitors as inducers of autophagic cell death. Epigenetic control of autophagy by methyltransferases has been reported in the setting of colorectal carcinoma where the methyltransferase EZH2 inhibits several negative regulators of mTOR (mechanistic target of rapamycin [serine/threonine]) leading to inhibition of autophagy [38]. Using drosophila models, Artal-Martinez de Narvajas and colleagues demonstrated that G9a binds to the promoter regions of autophagy-associated genes *LC3B*, *WIPI1*, and *DOR* and represses gene expression in a methyltransferase-dependent manner. Importantly, they showed that when T cells undergo glucose starvation, the G9a mono and di-methylation marks are removed from the promoter regions of these autophagy genes, resulting in their increased expression [39].

BIX01294, a G9a inhibitor, was identified as a chemical inducer of autophagic cell death through a chemical screen in a single engineered breast cancer cell line [40]. While the authors suggested that the mechanism was through ROS-induced autophagy, they did not provide extensive mechanistic evidence for this phenomenon. Similarly, other studies in oral squamous cell carcinoma [41] and neuroblastoma [42] have alluded to BIX01294-induced autophagy. None of these studies were done in T-ALL, however, and none explored whether this autophagic phenotype was reproducible across other G9a inhibitors or was unique to the BIX compound.

The SET domain-containing class of methyltransferase to which G9a belongs has been implicated in several metabolic diseases [43] and cellular states [44]. Although a direct role for G9a in glycogen metabolism in T-ALL has not been reported before, the notion that histone methyltransferases may be involved in metabolic regulation via transcriptional modulation has been postulated [22, 45]. Histone methylation and demethylation reactions depend on metabolic coenzymes like S-adenosylmethionine (SAM), flavin adenine dinucleotide (FAD) α-ketoglutarate (αKG) that are strictly connected with glycolysis and the tricarboxylic acid (TCA) cycle [46]. These metabolites fuel the one-carbon metabolic pathways that, in turn, use specific amino acids, i.e., threonine, serine, glycine, and methionine as one carbon donor-unit for macromolecules synthesis and ultimately cells proliferation. Recent findings suggest, for example, that *EHMT2* depletes serine and its downstream metabolites by repressing the transcription of genes involved in their biosynthesis such as phosphoglycerate dehydrogenase (*PHGDH*), phosphoserine aminotransferase 1 (*PSAT1*), phosphoserine phosphatase (*PSPH*), and serine hydroxymethyltransferase 2 (*SHMT2*) leading to a growth arrest in cancer cell lines of different tissue origins [22]. Our transcriptional analysis also suggests a similar modulation of one-carbon metabolism pathways and the suppression of key enzymatic regulators of the serine-glycine metabolism including *PHGDH*, *PSPH, SHMT2* and methylenetetrahydrofolate dehydrogenase/cyclohydrolase (*MTHFD2*). Interestingly, suppression of the serine hydroxymethyltransferase 1 and 2 (*SHMT1-2*) inhibits the progression of T-ALL in preclinical in vivo models [47], suggesting the need of improvement of different strategies -selective inhibition or epigenetic based- converging on one-carbon metabolism for the treatment of T-ALL.

Our study suggests that *EHMT2* is highly expressed in T-ALL and that they do not tolerate the loss of G9a activity. In response to the reduction of the glycolysis rate, T-ALL induce autophagy for survival to avoid cell death due the metabolic exhaustion [48]. Consistent with a *EHMT2/*G9a knockout *Drosophila* model where fly stocks accumulate glycogen [49, 50], we have observed an increase in glycogen-filled autophagosomes consistent with GSK-3 inhibition and derailed autophagy leading to cell apoptosis. *SESN2* contributes to these events by sustaining the inhibition of both ATP generating pathways: glycolysis and oxidative phosphorylation. Similarly, Ding and colleagues demonstrated that sestrin2 inactivation sensitizes cells to cell death associated with a rapid decline in the ATP level bypassing the canonical caspase-dependent, ROS and mTORC1-mediated apoptosis in lung adenocarcinoma models [27].

GSK-3 recently emerged as a potential therapeutic target in T-ALL [31, 51]. Tosello et al. demonstrated that 6-bromoindirubin-3'-oxime (BIO) inhibits GSK-3 and promotes apoptosis. They proposed that GSK-3 acts as an oncogene in T-ALL by promoting the stabilization of proteins such as MCL1 and c-MYB and XIAP. Furthermore, Hinze and colleagues showed that GSK3α inhibition profoundly sensitized drug-resistant leukemias to asparaginase by phenocopying a Wnt pathway activation signal [51]. Thus, a potential new strategy to exploit our observations will be testing the synergistic effect or identify effective combination strategies of G9a and GSK-3 inhibitors in T-ALL.

Poor pharmacokinetic/pharmacodynamic properties of available G9a drugs have limited their clinical development as potential HMT targets in cancer. However, promising lead compounds are emerging such as EZM8266 developed by Epizyme for sickle cell disease and DMX8.1 developed by the collaborative effort of the QIMR Berghofer Medical Research Institute and Domainex for solid tumors.

In conclusion, drawing on insights from genetic and chemical screens, these results suggest that G9a is an actionable dependency in T-ALL and provide further support for the anti-proliferative effects of suppressing G9a HMTase activity in T-ALL. Using a combination of immunocytochemical and ultrastructural analysis, we show that G9a/GLP inhibitors cause a rapid transition to late apoptosis and autophagic phenotypes in T-ALL. Importantly, we demonstrate a previously unexplored role of *EHMT2* in the control of glycogen metabolism in T-ALL, by modulating the metabolic sensor *SESN2* and highlight a need for further investigations into the role of methyltransferases in the epigenetic control of metabolism.

## Materials and methods

The complete description of the methods reagents is available in the Supplemental Materials and Methods.

### Primary T-ALL samples and T-lymphocytes

Primary patient leukemic cells or tissue biopsies were obtained under an approved protocol at the Parma University Hospital (n.18249/18/05/2017, n.265/2019) and according to the declaration of Helsinki guidelines for the protection of human rights.

### Seahorse XFp glycolytic rate assay

The glycolysis in live cells was measured with the Seahorse XFp Glycolytic Rate Assay (Agilent Technologies, #103346-100) and XFp Analyzer that directly measures real time extracellular acidification rates (ECAR) and oxygen consumption rate (OCR).

### Histone PTM quantification

Multiplexed PTM histones (beads) quantification was determined by a five point 2.5-dilution series of the samples normalizing for the level of H3 and detected with a Luminex MagPix instrument at Active Motif (Carlsbad, CA, USA).

### T-ALL in vivo studies

Non-obese diabetic, severe combined immune-deficient, interleukin (IL)-2 receptor gamma-deficient mice (NSG) were maintained in pathogen-free facilities at the University of Perugia. Local IACUC approved all procedures. T-ALLs cell, from previously established models (PDLX-0221), were injected into the retro-orbital venous sinus in adult (12–16 weeks old) sublethally-irradiated (0.9 Gy). PDLX-0121 (slowly grower), *TCRB*-*MYC*/t(7;8)(q34;q24) rearrangement, *CDKN2AB*/9p21 biallelic deletion, *PTEN*/10q24 deletion, received 26 doses of UNC0642 at 5 mg/kg every 48 starting 5 days post-transplant (euthanized 70 days post-transplant). PDLX-0122, (fast grower), *TRB*-*HOXA*/inv(7)(p15q34), *PTPN2*/18p11 deletion, *TP53*/17p13 deletion, *MYC*-translocation/t(8;?)(q24;?), received for nine doses UNC0642 at 5 mg/kg every 48 h starting 1-day post-transplant. CD1 mice were bred in the Animal Facility of the University of Parma and used to isolate thymic cells. Procedures were performed in accordance with European Community Directive 2010/63/UE and approved by the Ethics Committee of the University of Parma (Prot. 51/OPBA/16).

### Reporting summary

Further information on research design is available in the Nature Research Reporting Summary linked to this article.

## Supplementary information

Supplementary Figures

Supplemental Material and Methods

Supplementary Table 1

Supplementary western blot original file

Reporting Summary

## Data Availability

All data needed to evaluate the conclusion of this work are present in the paper. Uncropped western blots are presented as Supplemental Material. Additional information related to this manuscript may be requested from the corresponding author.
