# Supplementary material for: Identification of an Epi-metabolic dependency on EHMT2/G9a in T-cell acute lymphoblastic leukemia
Source: Cell Death Dis. 2022 Jun 17;13(6):551. doi: 10.1038/s41419-022-05002-5 (PMC9203761; doi:10.1038/s41419-022-05002-5)
Supplement: Supplementary file 1 — Supplementary Figures [file 41419_2022_5002_MOESM1_ESM.pdf]

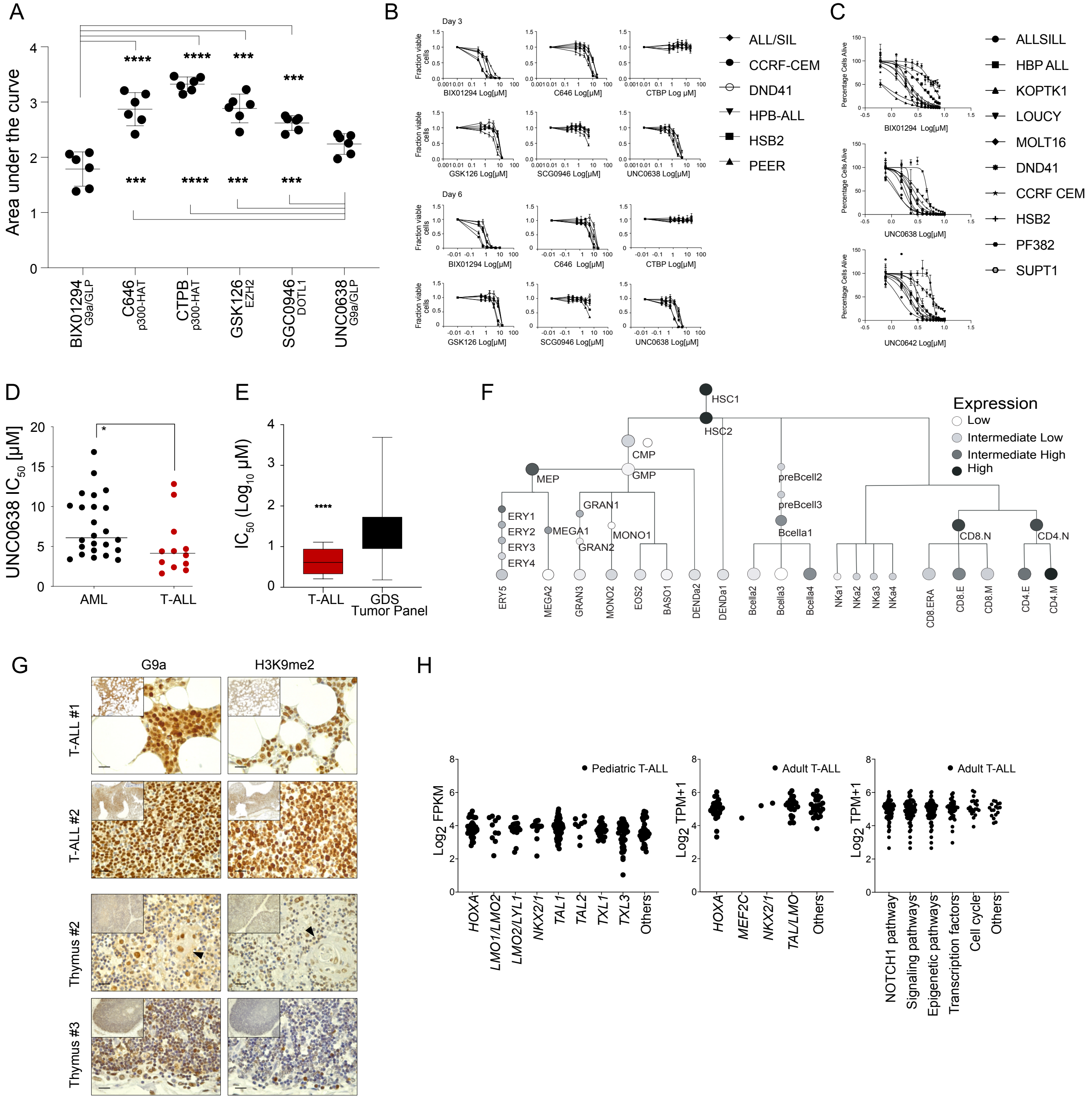

Supplementary Figure 1: (**A**) Effect of epigenetic modulators BIX01294, C646, CTPB, GSK126, SGC0946, and UNC0638 in T-ALL cell lines (ALL/SIL, CCRF-CEM, DND41, HPB-ALL, HSB2, and PEER) after three days of treatment. The scatter dot plot represents the effect of small molecules on cellular viability calculated using the area under the curve (AUC) model of log transformed dose-responses data using GraphPad V7. A lower AUC corresponds to a greater sensitivity. (**B**) Effect of BIX01294, C646, CTPB, GSK126, SGC0946, and UNC0638 in T-ALL cell lines (listed) on cell viability after three or six days of treatment. Error bars represent mean ± SD of three replicates. Graphs show fraction of viable cells relative to DMSO control as assessed by a CellTiter-Glo luminescence assay. (**C**) Effect of BIX01294, UNC0638, and UNC0642 in T-ALL cell lines (listed) on cell viability after three days of treatment. Error bars represent mean ± SD of three replicates. Graphs show fraction of viable cells relative to DMSO control as assessed by a CellTiter-Glo luminescence assay. (**D**) Dotplot showing response (IC_{50}) to the G9a inhibitor UNC0638 in T-ALL or AML cell lines screened as part of the Genomics of Drug Sensitivity in Cancer Project (GDS). (**E**) Whisker plot showing response (IC_{50}) to the G9a inhibitor UNC0638 in over 600 cancer cell in GDS. T-ALL cell lines are indicated in red and all other cancer cell lines in black. The line in the box-and-whisker diagram represents the IC_{50} median. The upper edge (hinge) of the box indicates the 75th percentile of the data set, and the lower hinge the 25th percentile. The ends of the vertical line indicate the minimum and the maximum data values. Statistical significance among groups (****$P < 0.0001$) was determined by a non-parametric t-test (Mann-Whitney). (**F**) Expression of *EHMT2* in the hematopoietic Differentiation Map (DMAP) database [37]. A gray scale is applied for increased expression. Black circles on the lineage tree represent high *EHMT2* expression on a spectrum, while the pale grey circles show lower *EHMT2* expression. (**G**) G9a and H3K9me2 expression in formalin-fixed paraffin embedded thymus and bone marrow human leukemia samples. Scale bar 20 µm. Arrowheads point to Hassal's corpuscles. (**H**) *EHMT2* expression with respect to recurring genetic alteration in pediatric (PMID 28671688) and adult (PMID 29279377) T-ALL clinical samples.

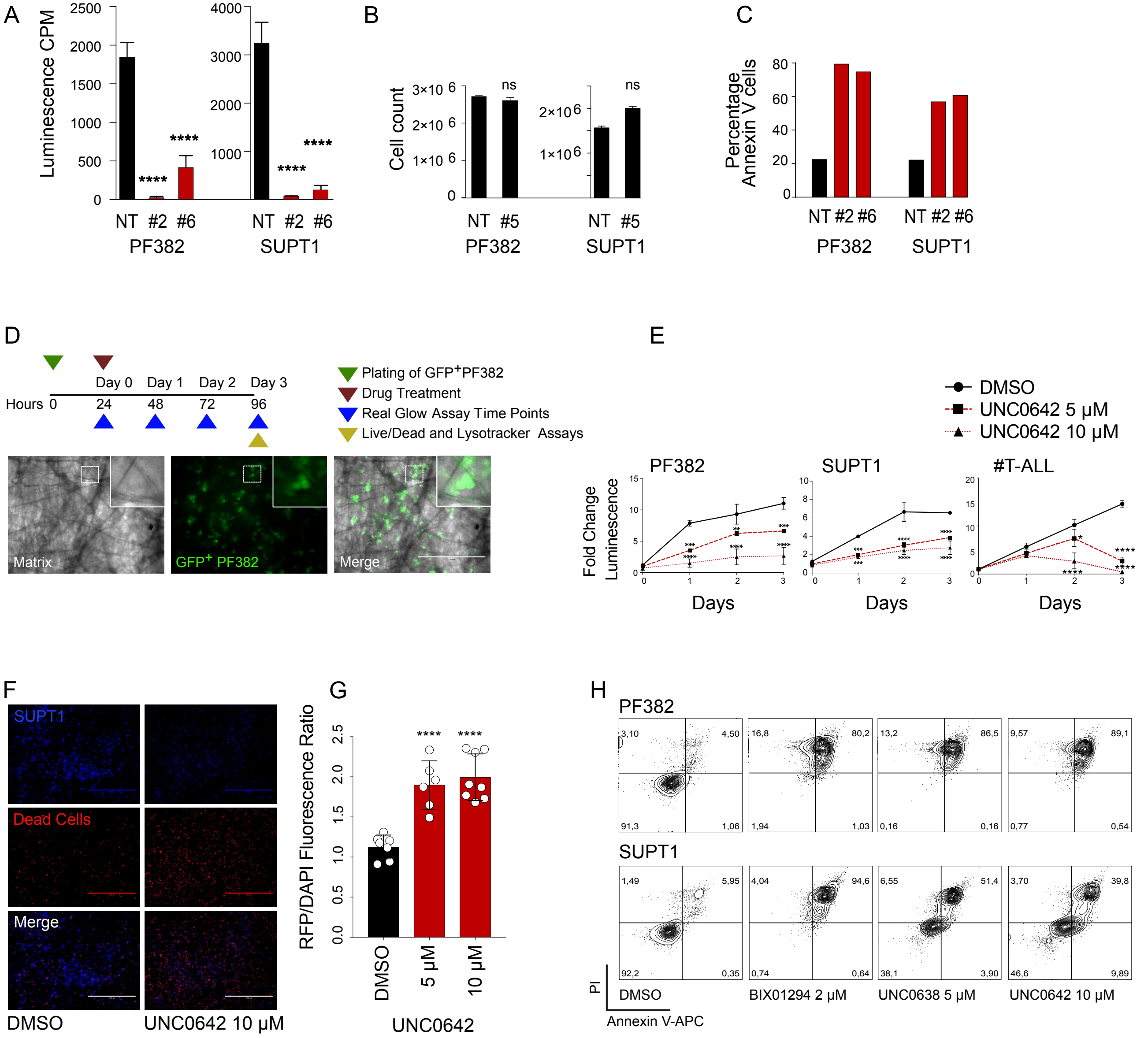

Supplementary Figure 2: (**A**) Effect of G9a knock down in PF382 or SUPT1 cells at six days post sgRNA selection. Histograms show ATP/ luminescence count per minute (CPM). Error bars denote the mean ± standard deviation (SD) of a minimum of three biological replicates. Statistical significance among groups (****$P < 0.0001$) was determined by a non-parametric t-test (Mann-Whitney). NT=non-targeting, #2 =sgRNA number 2, #6 =sgRNA number 6. (**B**) Effect of sgRNA #5 guide in PF382 and SUPT1 cells. Histograms show trypan exclusion assay absolute cellular count. Error bar denotes the mean ± SD of a minimum of three biological replicates. Statistical significance among groups (P=0.2 PF382, P=0.1 SUPT1 not significant) was determined by non-parametric t-test (Mann-Whitney). NT=non-targeting, #2 =sgRNA number 2. (**C**) Effect of G9a loss in PF382 and SUPT1 cells on induction of apoptosis. Annexin V/PI staining of PF382 and SUPT1 cells six days post sgRNA selection. Histograms show percentage of annexin V positive cells. NT=non-targeting, #2 =sgRNA number 2, #6 =sgRNA number 6. (**D**) Outline of the experiment (top of the panel). Representative picture of growing GFP positive (GFP+) PF382 cells on the inner polyester matrix of VITVO 3D bioreactor (bottom panel). (**E**) Cell viability assay of PF382 , SUPT1, and primary T-ALL cells in 3D culture treated with DMSO or UNC0642 at the indicated concentrations. T-ALL proliferation was assessed at the indicated time points using Real Time-GLO™ MT Assay and plotted as the luminescence (of the total colonized matrix) fold increase relative to Day 0. Error bars denote the mean ± standard deviation (SD) of three biological replicates. (**F**) Live Dead assay of SUPT1 (blue) cells growing in 3D cell culture treated with DMSO or UNC0642 at the indicated concentrations. Representative immunofluorescence images of control or UNC0642 treated SUPT1 upon a fluorescent based Live/Dead® assay staining at 72 hours. Cell death is indicated in the histogram (**G**) as a fluorescence ratio between DAPI (viable cells) and RFP (dead cells) signals of the acquired fields. Error bars denote the mean ± SD of one representative experiment. For imaging quantification, we selected fields with minor autofluorescence of the scaffold's matrix and no air bubbles. Images were captured by an EVOS FL microscope using Olympus long distance 4x objective (scale bar 1000 µm). Statistical significance among groups (*$P \leq 0.05$, **$P \leq 0.01$, ***$P \leq 0.001$, ****$P < 0.0001$) for all the experiments was determined by one-or two-way ANOVA (using Bonferroni's correction for multiple comparison testing). (**H**) Effect of G9a inhibitors treatments on induction of apoptosis. Annexin V/PI staining of T-ALL cells following 48 hours of treatment with the indicated concentrations of BIX01294, UNC0638, and UNC0642. A minimum of 20,000 events was collected for each condition.

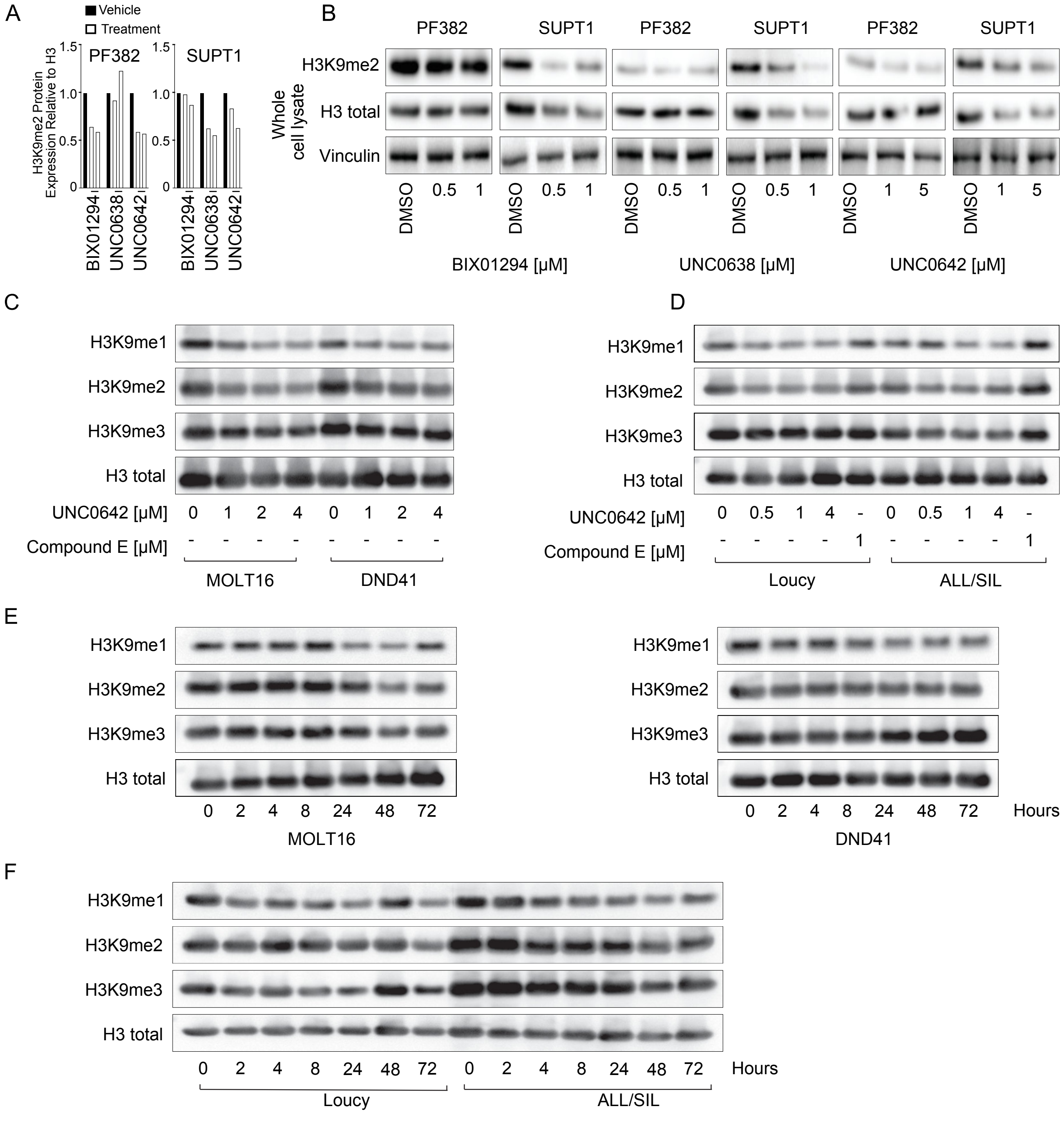

Supplementary Figure 3: (**A**) H3K9me2 expression relative to H3 from histone extracted G9a inhibitors treated cells. (**B**) Western blot showing expression of H3K9me2 in PF382 and SUPT1 cells treated at the indicated concentrations of G9a inhibitors for 48 hours. Protein lysates were obtained using a whole cells extraction protocol and stained with an antibody recognizing the H3K9me2 residue or total H3 with vinculin used as a loading control. (**C**) Western blot showing expression of H3K9me1, H3K9me2, and H3K9me3 in MOLT16 and DND41 cells treated at the indicated concentrations of UNC0642 for 48 hours. Protein lysates were obtained using an acidic histone extraction protocol, stained with an antibody recognizing the H3K9me1-3 residues. Total H3 was used as a loading control. (**D**) Western blot showing expression of H3K9me1, H3K9me2, and H3K9me3 in Loucy and ALL/SIL cells treated at the indicated concentrations of UNC0642 or Compound E for 48 hours. Protein lysates were obtained using acidic histone extraction protocol and stained with an antibody recognizing the H3K9me1-3 residues. Total H3 was used as a loading control. (**E**) Western blot showing time course dependent expression of H3K9me1, H3K9me2, and H3K9me3 in MOLT16 and DND41 cells treated with UNC0642 1 µM. Protein lysates were obtained using an acidic histone extraction protocol and stained with an antibody recognizing the H3K9me1-3 residues. Total H3 was used as a loading control. (**F**) Western blot showing time course dependent expression of H3K9me1, H3K9me2, and H3K9me3 in Loucy and ALL/SIL cells treated with UNC0642 1 µM. Protein lysates were obtained using an acidic histone extraction protocol and stained with an antibody recognizing the H3K9me1-3 residues. Total H3 was used as a loading control.

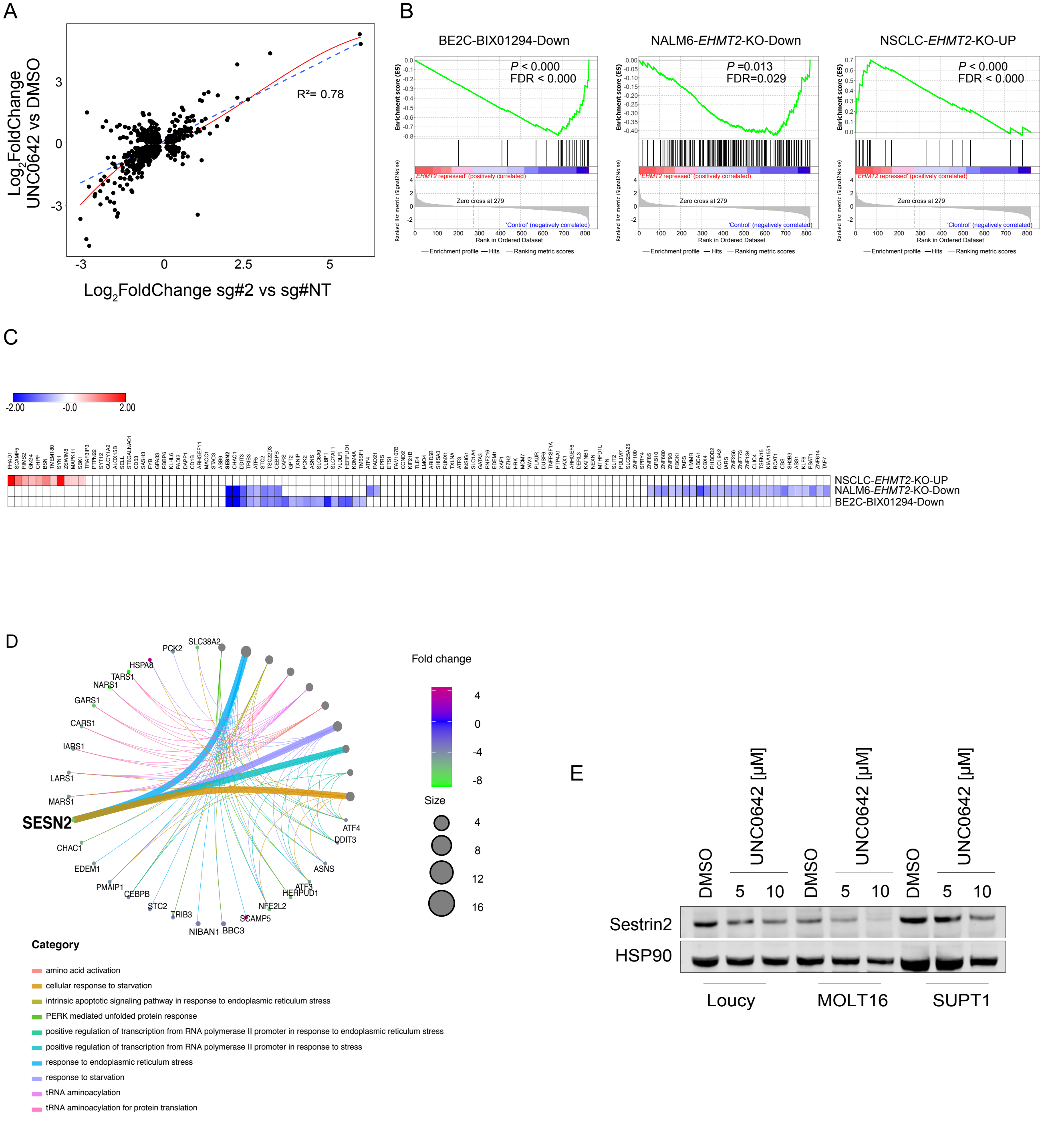

Supplementary Figure 4: (**A**) Dot plot showing significant gene expression changes in sg#2 versus sgNT or treated either with vehicle or UNC0642 (P < 0.05 and Log_2 fold changes >1.5) in PF382 cells. Pearson linear regression model is shown. (**B**) GSEA plot showing the enrichment of the genes down regulated by *EHMT2* in the two representative G9a "off" gene sets (BIX01294 left panel; *EHMT2* CRISPR-Cas9 knock down center and right panel). (**C**) Leading edge analysis of enriched genes. In the grid, the rows represent genes that constitute the main enrichment, leading edge (blue for down-regulated genes, red for up-regulated genes), in the different gene sets (column). (**D**) Circular net plot showing the network of differentially expressed genes in the first ten most strongly associated Gene Ontology (GO) processes. The size of each gray nodes represents the number of overlapped genes in each term and the colored scale bar represents the Log_2 fold change of each differentially expressed gene. (**E**) Western blot showing expression of sestrin2 in PF382 and SUPT1 cells 48 hours post G9a inhibitors treatment. Protein lysates were stained with an anti-sestrin2 antibody. HSP90 was used as loading control.

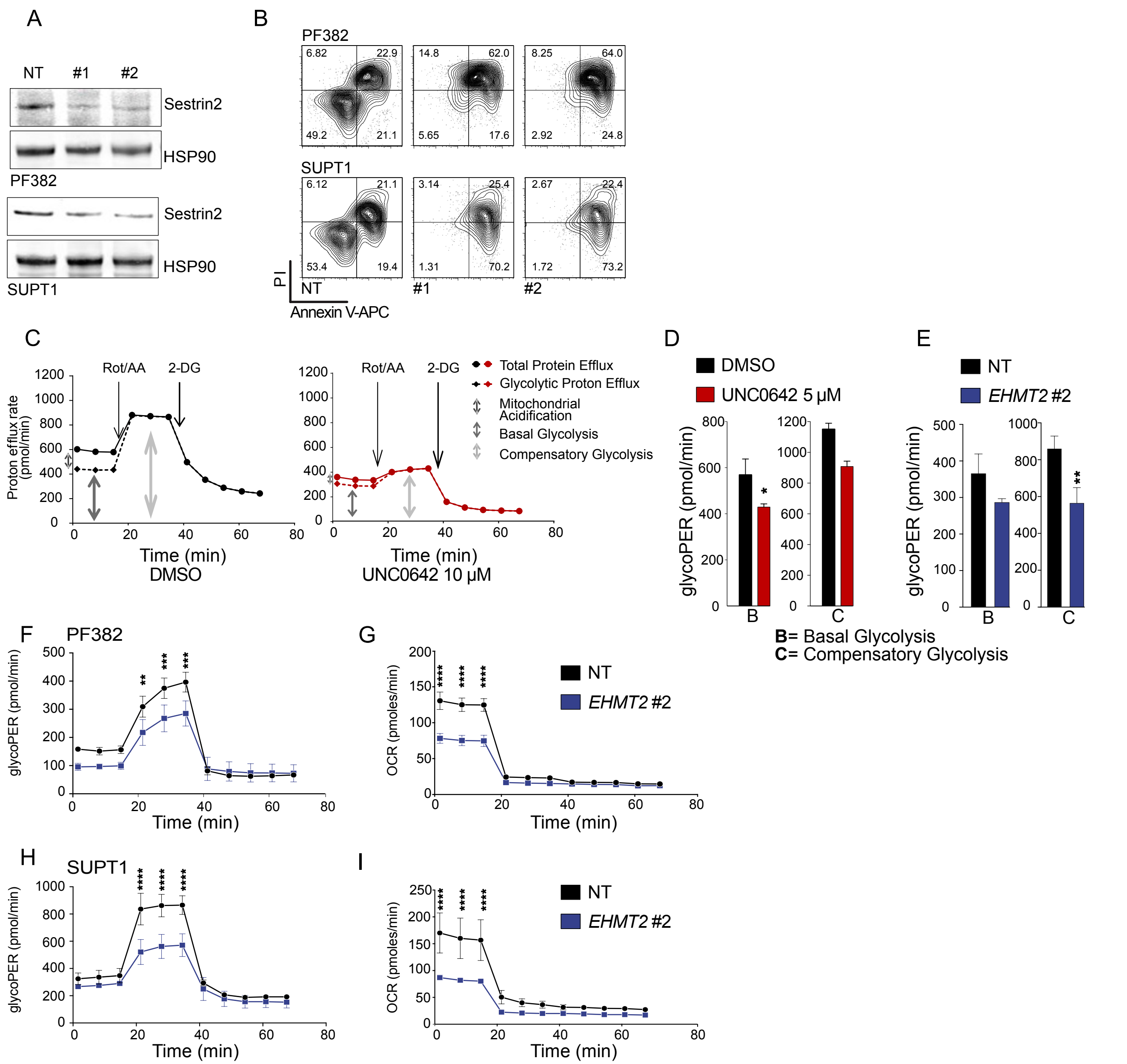

Supplementary Figure 5: (**A**) Western blot showing expression of sestrin2 in PF382 and SUPT1 cells two days post shRNA selection. Protein lysates were stained with an anti-sestrin2 antibody. HSP90 was used as loading control. NT=non-targeting, #1 or #2 =sestrin2 shRNA number 1 or 2. (**B**) Effect of *SESN2* repression in PF382 and SUPT1 cells on induction of apoptosis. Annexin V/PI staining of PF382 and SUPT1 cells five days post shRNA selection. 20,000 events were collected for each condition. (**C**) Representative Glycolytic Rate Assay profiled in PF382 cells treated with either DMSO or UNC0642. Proton efflux from live cells comprises both glycolytic and mitochondrial derived acidification. Inhibition of mitochondrial function by injecting rotenone & antimycin A (Rot/AA) enables calculation of mitochondrial-associated acidification. Subtraction of mitochondrial acidification from Total Proton Efflux Rate (PER) results in Glycolytic Proton Efflux Rate. The second injection is 2-deoxy-D-glucose (2-DG), which inhibits glycolysis. The resulting decrease in proton efflux rate provides qualitative confirmation that the PER produced prior to the injection is primarily due to glycolysis. (**D**) Glycolytic phenotype in PF382 cells. Histograms represent mean ± SD of three replicates of PF382 T-ALL cells treated with DMSO and UNC0642 (5 μM) on the x-axis. The y-axis represents the glycolytic proton efflux rate (PER) at the basal and compensatory level. Statistical significance among groups (*$P < 0.05$) was determined by a non-parametric t-test (Mann-Whitney). (**E**) Glycolytic phenotype in SUPT1 cells. Histograms represent mean ± SD of three replicates of SUPT1 T-ALL cells after *EHMT2* knock down on the x-axis. The y-axis represents the glycolytic proton efflux rate (PER) at the basal and compensatory level. Statistical significance among groups (**$P < 0.01$) was determined by a non-parametric t-test (Mann-Whitney). (**F-H**) Time resolved glycolytic proton efflux rate (glycoPER). Traces represent the mean ± SD of three replicates of *EHMT2* knock down PF382 (**F**) and SUPT1 (**H**) T-ALL cells analyzed over 70-minute time course experiments. Statistical significance among groups for #2 sgRNA guide vs. non-targeting (NT) (**$P < 0.01$, ***$P < 0.001$, ****$P < 0.0001$) was determined by two-way ANOVA using Bonferroni's correction for multiple comparison testing. (**G-I**) Oxygen Consumption Rate (OCR). Traces represent the mean ± SD of three replicates of *EHMT2* knock down PF382 (**G**) and SUPT1 (**I**) T-ALL cells analyzed over a 70-minute time course experiment. Statistical significance among groups for #2 sgRNA guide vs. non-targeting (NT) (****$P < 0.0001$) was determined by two-way ANOVA using Bonferroni's correction for multiple comparison testing.

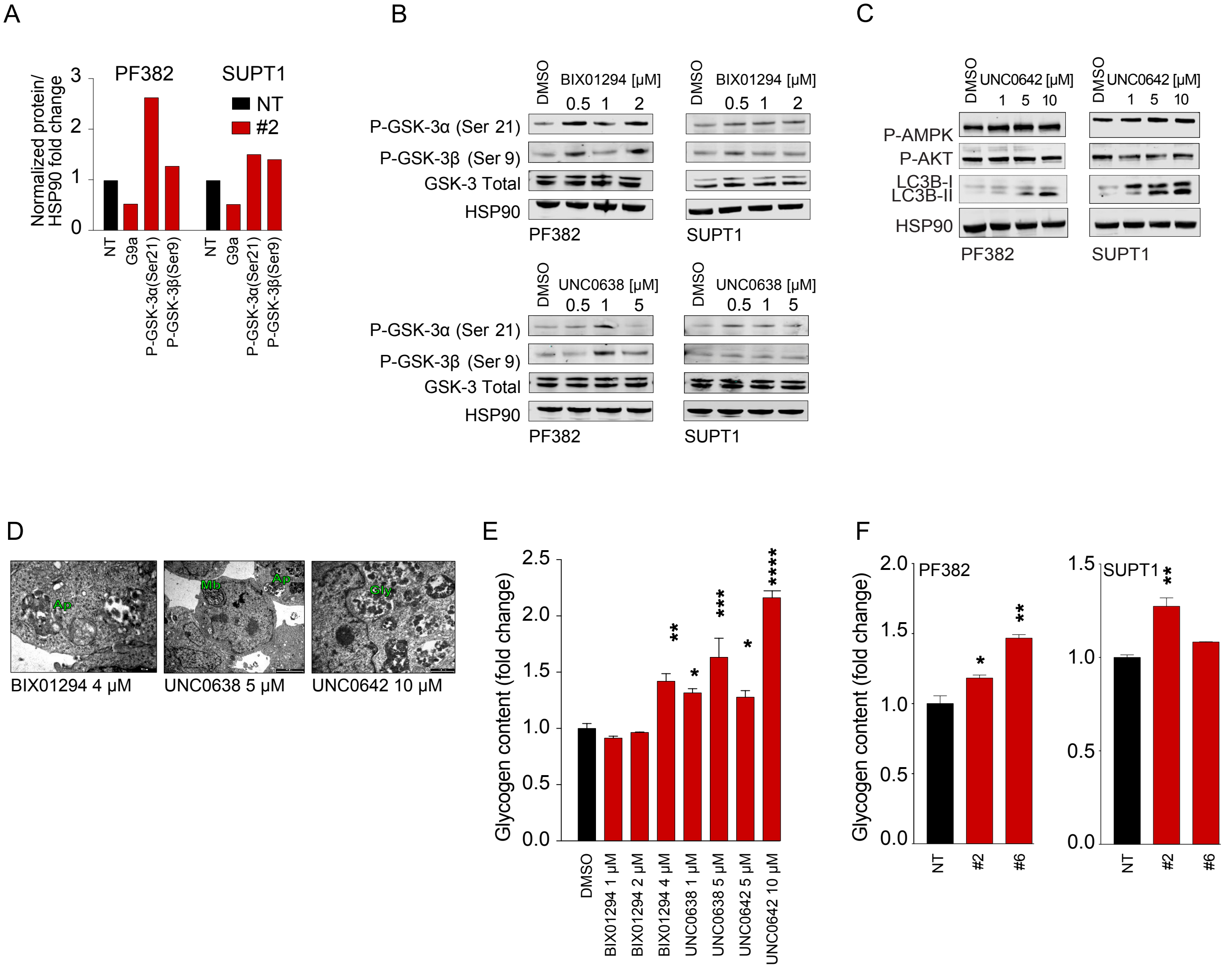

Supplementary Figure 6: (**A**) Densitometric quantification of indicated proteins after two days post sgRNA selection in PF382 and SUPT1 cells. The relative intensity of G9a/*EHMT2*, P-GSK-3α (Ser21) and P-GSK-3β (Ser9) was normalized for the levels of HSP90 and expressed as fold change relative to non-targeting (NT) sgRNA guide. (**B**) Modulation of GSK-3 by the G9a inhibitor BIX01294 and UNC0638. Western blot showing phosphorylation of serine-9 in GSK-3β or serine-21 in GSK-3α in T-ALL. Cell lysates were obtained after 48 hours of treatment. Protein lysates were stained with P-GSK-3α (Ser21), P-GSK-3β (Ser9), or GSK-3 Total. HSP90 was used as a loading control. (**C**) Modulation of p-AMPK and P-AKT by the G9a inhibitor UNC0642 in T-ALL. Western blot showing phosphorylation of P-AMPK and p-AKT in T-ALL. Cell lysates were obtained after 48 hours of treatment. Protein lysates were stained with P-AMPK, P-AKT, and LC3B. HSP90 was used as a loading control. (**D**) Transmission Electron Microscopic (TEM) images of T-ALL cells exposed for 48 hours to G9a inhibitors. Autophagosomes (Ap) displaying multilamellar bodies (Mb) or compartmentalization of electron dense material corresponding to glycogen granules (Gly). Scale Bars: left 0.5 µm; center 2 µm; right 1 µm. For DMSO see micrographs in Figure 6A and Supplementary Figure 7A. (**E**) Effect of G9a inhibitors BIX01294, UNC0638, and UNC0642 on cellular glycogen content. Histograms show the glycogen fold change increase relative to a DMSO control after G9a inhibitors treatment for 48 hours in the SUPT1 cell line. Error bars denote the mean ± standard deviation (SD) of two biological replicates. Statistical significance among groups (*$P \leq 0.05$, **$P \leq 0.01$, ***$P \leq 0.001$, ****$P < 0.0001$) was determined by one-way ANOVA (using Bonferroni's correction for multiple comparison testing). (**F**) Effect of G9a knock down on cellular glycogen content. Histograms show the glycogen fold change increase relative to a non-targeting (NT) G9a in PF382 and SUPT1 cell lines. Error bars denote the mean ± standard deviation (SD) of two biological replicates. Statistical significance among groups (*$P \leq 0.05$, **$P \leq 0.01$) was determined by one-way ANOVA (using Bonferroni's correction for multiple comparison testing).

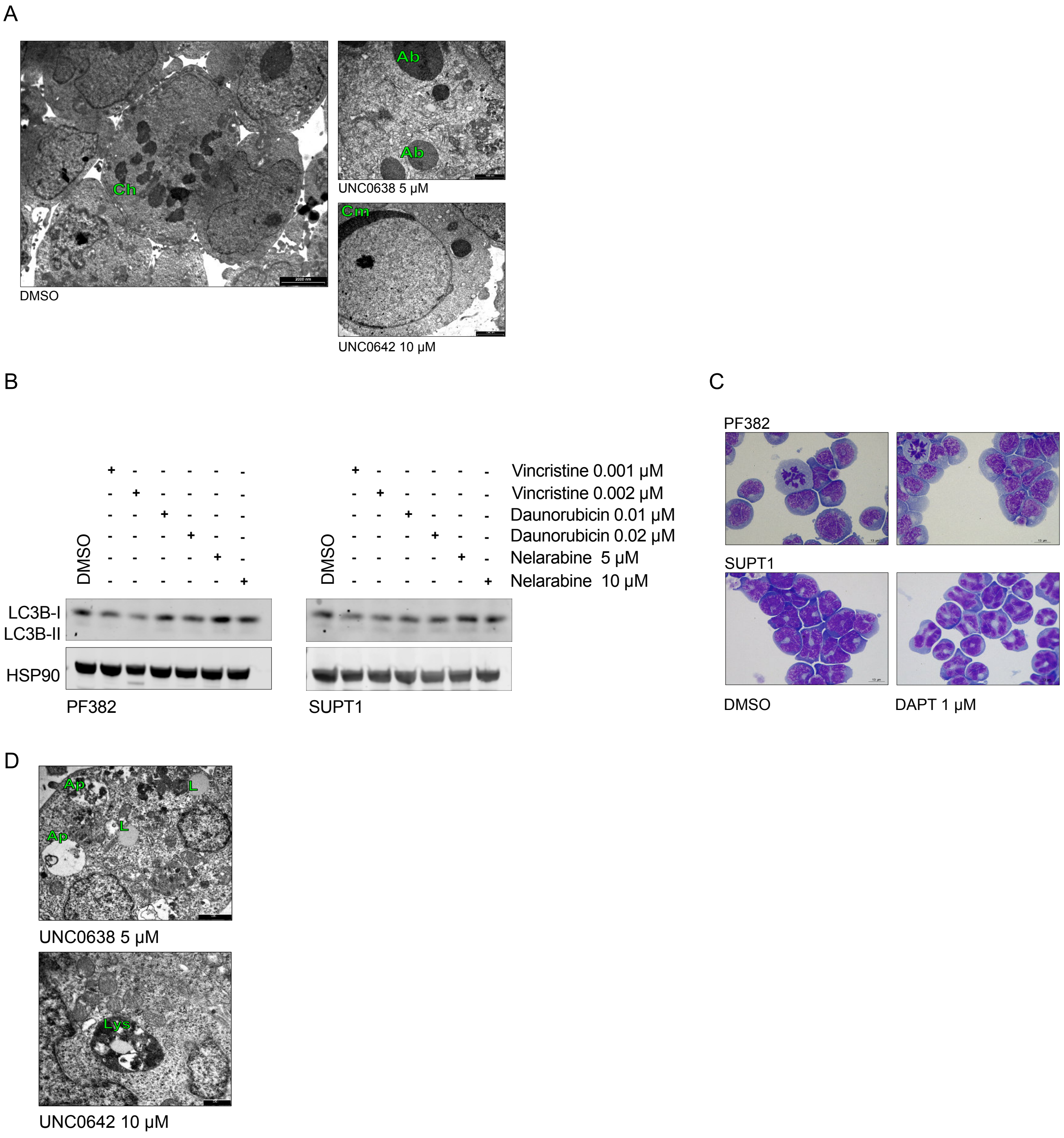

Supplementary Figure 7: (**A**) Transmission Electron Microscopic (TEM) image of untreated (DMSO) T-ALL cells one of which shows mitotic chromosomes (Ch). Scale bar: 2 µm. The two right panels illustrate cultures of the PF382 cell line exposed for 48 hours to the indicated concentrations of different G9a inhibitors. Apoptotic features are documented by round cytoplasmic apoptotic bodies (Ab, upper) and a nucleus undergoing DNA degradation and chromatin margination (Cm, lower). Scale bars: 1 µm. (**B**) Western blot showing expression of LC3B-I and LC3B-II in PF382 and SUPT1 cells treated at with vincristine, daunorubicine or nelarabine at the indicated concentrations after 48 hours. HSP90 was used as a loading control. (**C**) Representative images of May Grunwald-Giemsa stained cytospin preparations of T-ALL (PF382 and SUPT1) cultured in vehicle (DMSO) or DAPT for 48 hours. Images were captured with a Leica ICC50W optical microscope (100X). Scale bars: 13 µm. (**D**) High magnification TEM images of G9a inhibitors-treated T-ALL cells to illustrate the ultrastructural characteristics of autophagosomes (Ap) and the presence of free lipid droplets (L) or engulfed by phagolysosomes (Lys). Scale Bars: top 1 µm; bottom 0.5 µm.

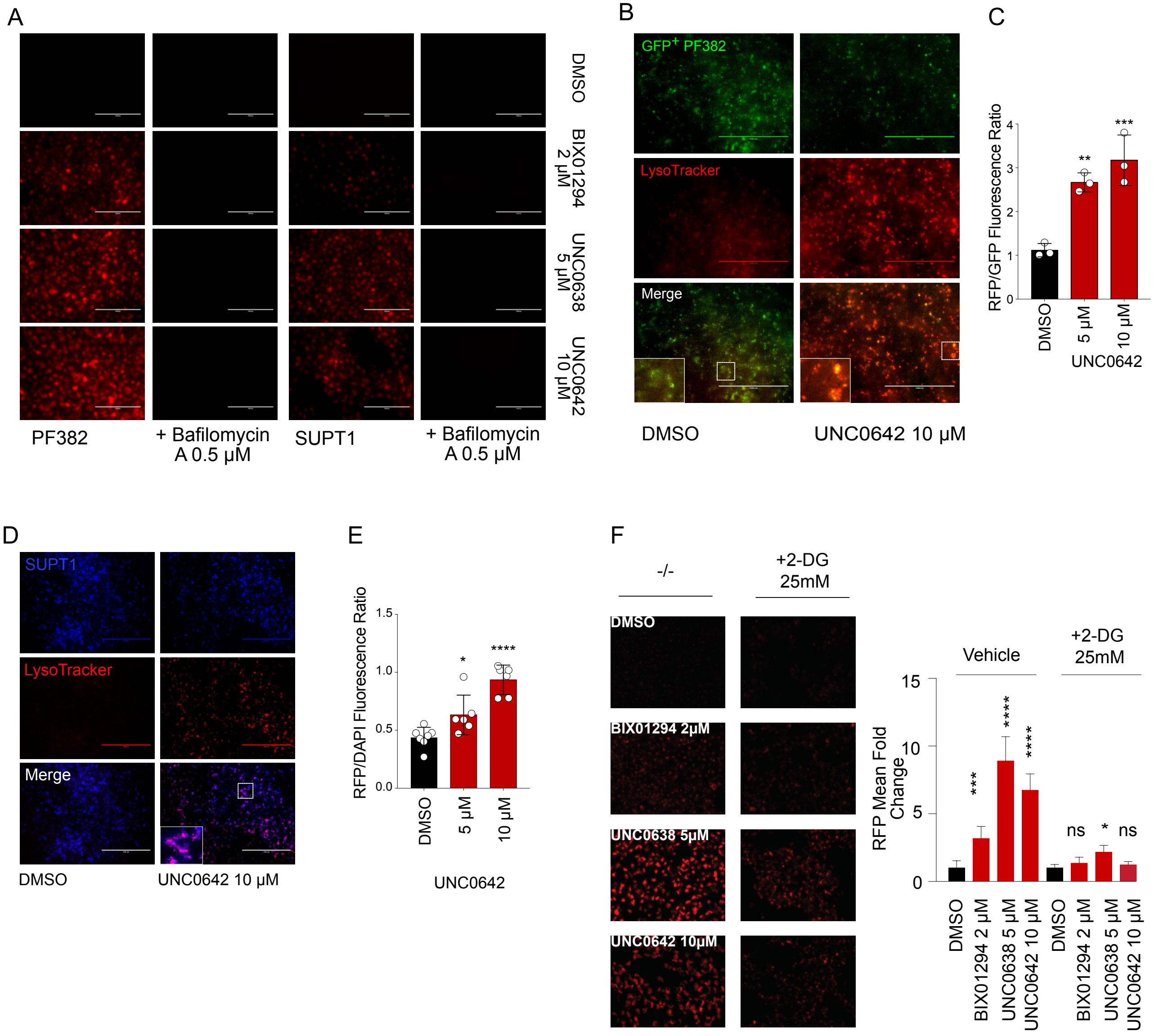

Supplementary Figure 8: (**A**) LysoTracker® Red DND-99 staining of T-ALL cells treated at the indicated concentrations of G9a inhibitors and 0.5 μM Bafilomycin A for 48 hours. Scale bars: 100 μm. (**B**) Induction of autophagy in UNC0642 treated PF382 growing in 3D bioreactors. Autophagosome/lysosome formation was evaluated by the fluorescence based LysoTracker assay. Images were captured by EVOS FL microscope using Olympus long distance 4x objective. Scale bar: 1000 μm. (**C**) Induction of autophagy in UNC0642 treated PF382 growing in 3D cell culture bioreactor. Autophagosome/lysosome formation was evaluated by the fluorescence based and quantified as fluorescence ratio between RFP (acidic vesicles) and GFP (viable cells) signals of the acquired fields (on the bottom). Error bars denote the mean ± standard deviation (SD) of one representative experiment. For imaging quantification, we selected fields with minor autofluorescence of the scaffold's matrix and no air bubbles. (**D**) Induction of autophagy in UNC0642 treated SUPT1 cells growing in a 3D cell culture bioreactor. Autophagosome/lysosome formation was evaluated by the fluorescence based LysoTracker assay and quantified (**E**) as fluorescence ratio between RFP (acidic vesicles) and blue (viable cells) signal per acquired fields. Error bars denote the mean ± standard deviation (SD) of one representative experiment. For imaging quantification, we selected fields with minor autofluorescence of the scaffold's matrix and no air bubbles. Images were captured by an EVOS FL microscope using Olympus long distance 4x objective (scale bar 1000 μm). Statistical significance among groups (*$P \leq 0.05$, **$P \leq 0.01$, ***$P \leq 0.001$, ****$P < 0.0001$) for all the experiments was determined by one- or two-way ANOVA (using Bonferroni's correction for multiple comparison testing). (**F**) LysoTracker® Red DND-99 labeled T-ALL cells treated with G9a inhibitors in the presence or absence of 25 mM 2-Deoxy- d-glucose (2-DG). Scale Bars: 100 μm. Histograms show the mean and ± SD of RFP per field (n=9) relative to the vehicle control. Statistical significance among groups (*$P \leq 0.05$, ***$P \leq 0.001$, ****$P < 0.0001$) was determined by one-way ANOVA (using Bonferroni's correction for multiple comparison testing).
