# Supplementary material for: Identification of an Epi-metabolic dependency on EHMT2/G9a in T-cell acute lymphoblastic leukemia
Source: Cell Death Dis. 2022 Jun 17;13(6):551. doi: 10.1038/s41419-022-05002-5 (PMC9203761; doi:10.1038/s41419-022-05002-5)
Supplement: Supplementary file 2 — Supplemental Material and Methods [file 41419_2022_5002_MOESM2_ESM.docx]

**MATERIALS AND METHODS**

**Cell Culture**

Human cell lines SUPT1, MOLT16, LOUCY, DND41, HPB-ALL, and MOLT4 were purchased respectively from Leibniz-Institut DSMZ-German collection of microorganisms and cell cultures (Germany) and American Type Culture Collection (ATCC, Manassas, VA, USA); identities of ALL/SIL, KOPTK1, PF382, and SUPT11, were confirmed by PCR sequencing for known *NOTCH1* mutations and short tandem repeat (STR) loci profiling. CCRF-CEM, HSB2 and PEER were a gift from the Bradner lab (Dana-Farber Cancer Institute, Boston, MA, USA). HS-5 cells were a gift from Giuliani lab (University of Parma. Cells were cultured in RPMI 1640 or DMEM (Thermo Fisher Scientific, Waltham MA, USA, Waltham MA, USA #MT10040CV) with 10% fetal bovine serum (FBS) (Sigma-Aldrich, St. Louis, MO, USA, #F2442-500ML) and 1% penicillin-streptomycin (Thermo Fisher Scientific, Waltham MA, USA, #3MT30002CI) and incubated at 37°C with 5% CO_2_.

**Primary T-ALL Samples, T-lymphocytes and Murine Thymic cells**

Primary patient leukemic cells or tissue biopsies were obtained under an approved protocol at the Parma University Hospital (n.18249/18/05/2017, n.265/2019) and according to the declaration of Helsinki guidelines for the protection of human rights. PB and BM samples were collected at the time of diagnosis. Mononuclear cells were isolated by density gradient centrifugation using LSM-lymphocyte separation medium (Cappel^TM^ MP Biomedicals, LLC, Ohio, USA #50494). Lymphocytes were isolated from PBMC by using a CliniMACS Prodigy (Miltenyi Biotec, Bergisch Gladbach, Germany) and cultured for a short time using the same growth conditions described above. Cells were seeded in 384-well plates (Corning Life Sciences Plastic, Bedford MA, USA, #3570) at the final concentration of 0.02x10^6^/mL per condition. Small molecules were added with a nanometric dispenser Tecan D300e (Tecan Trading AG, Switzerland). Clinical samples were plated alive (trypan blue exclusion assay > 90% in all cases) and viability was assessed after 72 hours of drug treatment using a CellTiter-Glo ATP assay (Promega Corporation, Madison, WI, USA, #G7573). The analysis was performed using GraphPad Prism software (La Jolla, CA, USA) to calculate the IC_50_ and the areas under the curve (AUC) of drugs.

Murine thymic cells were isolated by enzymatic digestion (CorningTM RPMI-1640 with 1,5 µg/mL Collagenase/Dispase®, Roche, and 1 µl/ml Dnase I, ThermoFisher) 37 °C for 15 minutes. The supernatant fraction was collected in a 50 mL tube containing 10 mL of cold albumin-rich buffer (1X PBS [Ca2+/Mg2+-free] with 0,5 % Bovine serum albumin and 2mM ethylenediaminetetraacetic acid [EDTA]) to neutralize the enzymes. The operations were repeated 2 more times, until complete tissue digestion; then the pooled supernatants were centrifuged at 400 x g, 4 °C for 8 minutes. The pelleted cells were resuspended in 10 mL cold albumin-rich buffer and counted using a hemocytometer. The separation of the CD25^-^ cells was performed using the mouse CD25 MicroBead Kit (MACS Milteny Biotec) according to manufacturer’s instructions. Briefly, 2 x 10^8^ cells were resuspended in 1.96 mL of albumin-rich buffer, added with 40 µL of CD25-PE-conjugated antibody and incubated for 10 minutes at 4°C. After washing, the cells were centrifuged and resuspended in 1.8 mL of albumin-rich buffer before the addition of 200 µL of anti-PE MicroBeads, followed by 15 minutes of incubation at 4°C. After being further washed and centrifuged, pelleted cells were resuspended in 1 mL albumin-rich buffer. The cell suspension was applied onto a MS Column, previously assembled with a pre-separation filter and placed on the magnetic separator. The flow-through containing CD25^-^ cells was collected in a 15 mL tube. Then, the column was removed from the magnetic separator to flush out and collect the CD25^+^ cells in a separated tube for validation experiments.

**Cell Viability, Proliferation and Apoptosis**

ATP-based cell viability was determined using the CellTiter-Glo Luminescent Cell Viability Assay (Promega Corporation, Madison, WI, USA #G7573). Luminescence was measured using a Fluostar Omega instrument (BMG-labtech, Ortenberg, Germany) or a Victor X4 (Perkin Elmer, Waltham, MA, USA). Apoptosis was measured by Annexin V and propidium iodide (PI) staining using flowcytometry (Affymetrix, eBioscience, San Diego, CA, USA, 88-8007-74). Cells were analyzed by flow cytometry with a FACScan flow cytometer (BD, Franklin Lakes, NJ, USA or Beckman Culture-Cytomics FC 500, Life Sciences Division, Indianapolis, USA) and FlowJo V10 (Tree Star LLC, Ashland, OR, USA) analytical software.

Compounds were obtained from the following sources: BIX01294 (R&D Systems Inc., Minneapolis, MN, USA #3364/10mg, or Selleckchem, Houston, TX USA, #S8006/10mg); UNC0638 and UNC0642 were donated from Jian Jin (Icahn School of Medicine, Mt. Sinai, New York, NY) or purchased UNC0638 (Cayman Chemical, Ann Arbor, Michigan USA #1255580-76-7 or Selleckchem, #S8071); UNC0642 (Sigma-Aldrich, St. Louis, MO, USA, #SML1037 5 mg); compound E (ENZO Life Sciences, Farmingdale, NY, USA, #ALX-270-415-M001), vincristine sulfate (Tocris Bio-techne, Bristol, UK #1257); nelarabine (Tocris Bio-techne, Bristol, UK # 6359); daunorubicin (Selleckchem, Houston, TX USA, #S3035); GSK126 (Selleckchem, Houston, TX USA, #S7061); SGC0946 (Selleckchem, Houston, TX USA, #S7079), C646 (Cayman Chemical, Ann Arbor, Michigan, USA, #10549); CTPB (Sigma Aldrich-Merck division Darmstadt, Germany #EP001); Bafilomycin A1 (InvivoGen, San Diego, CA, USA CAS88899-55-2), SB216763 (Selleckchem, Houston, TX USA, #S1075), CHIR-99021 (Selleckchem, Houston, TX USA, #S1263).

**sgRNA Design**

CRISPR sgRNA were designed using the Broad Institute guide designer which implements the Rule Set 1 sgRNA scoring algorithm. Guides were specifically designed to target the enzymatic domain (SET) of *EHMT2*. The complete DNA sequence of Human *EHMT2* was analyzed using the NCBI gene ID database. Below are the guide sequences.

| *EHMT2* sgRNA | End | Oligonucleotide Sequence | Target |
| --- | --- | --- | --- |
| #2 | 5’ | CACCGGTGCAGCATGAAGACCCGGA | C-terminus SET domain |
| #2 | 3’ | AAACTCCGGGTCTTCATGCTGCACC | C-terminus SET domain |
| #5 | 5’ | CACCGTGGTTACACTCGAAAATCAG | Pre-SET domain |
| #5 | 3’ | AAACCTGATTTTCGAGTGTAACCAC | Pre-SET domain |
| #6 | 5’ | CACCGTTCGACTTAGACAACAAGGA | Interaction SET-H3 |
| #6 | 3’ | AAACTCCTTGTTGTCTAAGTCGAAC | Interaction SET-H3 |

**Plasmids, Cloning and Lenti-viral constructs**

Desalted oligonucleotides were synthesized by the Molecular Biology Core Facility (at Dana-Farber Cancer Institute, Boston, MA, USA). *EHMT2* target guide sequence cloning was done using the Lentiviral CRISPR tool box in the Genome-Scale CRISPR knock down protocol. CRISPR plasmids were digested using the FastDigest Esp11 (#FD0454), FastDigest Buffer (Thermo Fisher Scientific, Waltham MA, USA #B64) and Fast Alkaline Phosphatase (Life Technologies, Carlsbad, CA, USA, #EF0651). Digested plasmids were purified using the QIAquick Gel Extraction Kit (Qiagen, Hildberg, Germany, #28704). Oligos were annealed using T4 ligation buffer ((Fisher Scientific (Thermo Fisher Scientific), Waltham MA, #46300-018) and T4 PNK enzyme (supplemented with ATP), (New England Biolabs, Ipswich MA, USA, #M0201S). Transformation was done by heat-shock using One Shot Stbl3 Chemically Competent E. coli protocol (Life Technologies, Carlsbad, CA, USA, #C737303). Plasmid isolation was performed using the HiSpeed Plasmid Maxi Kit Qiagen (Qiagen, Hildberg, Germany, #12663).

*SESN2* PLKO.1 based shRNA vectors were obtained from MERK (Sigma-Aldrich, St. Louis, MO, #SHCLNG-NM-031459) and sequences are listed below.

| Catalog # | Oligos | shRNA number |
| --- | --- | --- |
| TRCN0000142035 | CCGGGCCCGAATCCTAGTTCAGTTTCTCGAGAAACTGAACTAGGATTCGGGCTTTTTTG | #1 |
| TRCN0000143630 | CCGGGAAGACCCTACTTTCGGATATCTCGAGATATCCGAAAGTAGGGTCTTCTTTTTTG | #2 |

cDNA-*SESN2*-orf were obtained from OriGene (SESN2 (NM_031459) Human Tagged ORF clone #RC201386).

**Virus Production and Transduction of T-ALL Cell Lines**

pCMV-VSV-G envelope vector and Delta 8.9 packaging plasmid constructs were used for making virus (Addgene, Cambridge MA, USA).  293T adherent were plated at 1.5 million/ml in DMEM media (Life Technologies, Carlsbad, CA, USA, # 11965118), 10% fetal bovine serum (FBS) (Sigma-Aldrich, St. Louis, MO, USA, #F2442-500ML) and 1% penicillin-streptomycin (Fisher Scientific (Thermo Fisher Scientific)), Waltham MA, #3MT30002CI) and incubated at 37°C with 5% CO_2_, until confluent.   DMEM media was aspirated from and replaced with 5 ml of RPMI media (with 10% FBS, 1% Penicillin Streptomycin). The transduction mix containing the entry and envelope vectors (VSVG/ Delta 8.9, CRISPR/Cas9 and sgRNA or shRNA constructs, FuGENE 6 Transfection Reagent (Promega Corporation, Madison WI, USA, #E2691) and Opti-MEM Reduced-Serum medium (1X) liquid (Life Technologies, Carlsbad, CA, USA, #31985062) was added to 293T cells. Virus mix was incubated at 37°C for 72 hours. Same day transduction of PF382 and SUPT1 cells was performed with non-frozen, freshly harvested virus as follows: 4 million cells were plated in 100 µl of RPMI media with polybrene in triplicate replicates in a round bottom 96 well plate and infected with 100 µl of virus. A double spin-infection was done at 2500 RPM for 2 hours at temperature of 25°C.  Cells were expanded at 24- and 48-hours post infection and selected in puromycin (1µg/ml) (Invitrogen, San Diego, CA, USA #ANT-PR-1) at the 72-hour time point.

**Protein Sample Preparation**

Whole cell protein lysate was extracted using 1X Cell Lysis buffer (Cell Signaling Technology, Danvers, MA, USA, #9803S) with Phospho-stop, Phosphatase Inhibitor (Sigma-Aldrich, St. Louis, MO, USA, #04906837001) or Protease/phosphatase Inhibitor Cocktail 100X (Cell Signaling Technology, Danvers, MA, USA, #58725) and Complete Mini, EDTA-free Protease Inhibitor (Sigma-Aldrich, St. Louis, MO, USA, #11836170001). Cytoplasmic and nuclear extractions were performed as described in the Nuclear Extract Kit (Active Motif, Carlsbad, CA, USA, #40010). The Abcam Histone Extraction Protocol was used for all histone extractions (Abcam, Cambridge, United Kingdom, #AB221031), with minor amendments. Cells were harvested and washed twice with ice-cold Phosphate Buffered Saline (PBS), then suspended in Triton Extraction Buffer (TEB: PBS containing 0.5% Triton X 100 (v/v), 2 mM phenylmethylsulfonyl fluoride (PMSF), 0.02% (w/v) NaN3). Cells were lysed on ice for 10 minutes with gentle stirring and centrifuged at 2000 RPM for 10 minutes at 4°C to spin down the nuclei. The supernatant was discarded.  Nuclei were washed in half the volume of TEB and centrifugation repeated as before. The pellet was then re-suspended in 0.2 Na HCl and left overnight at 4°C. Samples were centrifuged at 6,500 XG for 10 minutes at 4°C to pellet debris. Supernatant containing the histone protein was collected for analysis by western blot.

**Antibodies**

Knockout of *EHMT2* was confirmed by western blot of whole cell lysate using anti-G9a/EHMT2 (C6H3) rabbit monoclonal antibody (Cell Signaling Technology, Danvers, MA, USA, #3306S). Anti-GAPDH mouse monoclonal antibody (Santa Cruz Biotechnology, Dallas, TX, USA, #sc-47724) was used a loading control. For histone fractions the following antibodies were used: mouse monoclonal to histone H3 (di methyl K9) (Abcam, Cambridge, United Kingdom, #AB1220); rabbit polyclonal antibody to histone H3 (mono methyl K9) (Abcam, Cambridge, United Kingdom, #AB8896), rabbit polyclonal antibody to histone H3 (tri methyl K9) (Abcam, Cambridge, United Kingdom, #AB8898) . Anti-Histone 3 rabbit polyclonal antibody (total) was used as a loading control (Abcam, Cambridge, United Kingdom, #AB1791) and vinculin (Santa Cruz Biotechnology, Dallas, TX, USA, #sc-25336) was used as loading control for Whole cell lysate.

To assess the effect of G9a inhibitors on glycogen synthesis at the protein level, western blot of whole cell lysate was performed using the following GSK-3 antibodies listed below: rabbit monoclonal antibody phosho-GSK-3α (Ser21) (#36E9), rabbit monoclonal antibody phospho-GSK-3β (Ser9, #9336S), rabbit monoclonal antibody total GSK-3α/β (D75D3) (#5676S), rabbit polyclonal phospho-mTOR (Ser2448)(#2971), rabbit polyclonal phospho-p70 S6 Kinase(Thr389)(#9205), rabbit monoclonal antibody phosho-AMPKα (Thr172) (40H9) (#2535), and mouse monoclonal antibody phosho-Akt (Ser473) (587F11) (#4051) (Cell Signaling Technology, Danvers, MA, USA).

To confirm *EHMT2*/G9a expression at a protein level in T-ALL cell lines with different *NOTCH1* status, western blot was performed using G9a/EHMT2 (C6H3) rabbit monoclonal antibody (Cell Signaling Technology, Danvers, MA, USA, #3306S), EHMT1/GLP mouse monoclonal antibody (Abcam, Cambridge, United Kingdom, #AB41969) and Cleaved Notch1 (Val1744) (D3B8) rabbit monoclonal antibody (Cell Signaling Technology, Danvers, MA, USA, #4147).

Apoptosis and autophagy were assessed with the following antibodies: rabbit polyclonal antibody LC3B (#2775), sestrin2 (D1B6) rabbit monoclonal antibody (#8487) (Cell Signaling Technology, Danvers, MA, USA). HSP90 (4F10) mouse monoclonal antibody (Santa Cruz Biotechnology, Dallas, TX, USA, #sc-69703) and β-Actin (13E5) rabbit monoclonal antibody (Cell Signaling Technology, Danvers, MA, USA, #4970) were used as a loading control.

For chemiluminescence based development the following secondary antibodies were used: anti-mouse IgG, peroxidase-linked species-specific F (ab')2 fragment Fisher Scientific (Thermo Fisher Scientific, Waltham MA #45000680); anti-rabbit IgG, peroxidase-linked species-specific F(ab)2 fragment (from donkey), (Thermo Fisher Scientific, Waltham MA #45000680, MA #45000683). All fluorescent secondary antibodies used were obtained from LI-COR Biosciences, Lincoln, NE, USA. They include: IRDye 680LT Goat anti-Mouse IgG 0,1mg (#925-68020); IRDye 800CW goat anti-rabbit IgG 0,1mg (#925-32211); IRDye 680RD goat anti-rabbit IgG 0,1 mg (#925-68071).

**Western Blot**

Proteins were quantified using Bio-Rad Protein Assay Dye Reagent (Bio-Rad Laboratories, Hercules, CA, USA, #5000006). For western blot immuno-detection, we used the Renaissance Western Blot Chemiluminescence reagent with enhanced luminol (Perkin Elmer, Waltham, MA, USA, #NEL104001EA) and BIOMAX MR FILM (Maximum Resolution (Carestream Health Inc., Rochester, NY, USA, #870 1302). Western blot quantification is available as supplemental table.

**Glycogen Colorimetric Assay**

Glycogen concentration was measured using a Glycogen Colorimetric/Fluorometric Assay Kit (BioVision Inc., CA, USA, #K646-100) at 550 nm according to the manufacturer's manuals. One million cells were harvested following 48h treatment of DMSO or G9a inhibitors. Samples were homogenized with deionized water (5000 cell/µL). Denatured homogenates were centrifuged for 10 min at 15000 rpm, and supernatants were suspended with hydrolysis buffer and 50 μL were spotted in a 96-well plate. Plates were incubated for 30 min at room temperature in the presence of a reaction buffer until glycogen was measured using a Victor X4 (Perkin Elmer, Waltham, MA, USA) at 550nm. Data were normalized to cell number.

**Transmission Electron Microscopy**

Pellets of PF382 cells treated with DMSO, BIX01294 4 µM, UNC0638 5 μM or UNC0642 10 μM were fixed in Karnovsky solution (4% formaldehyde, 5% glutaraldehyde) for 90 minutes at room temperature. Cells were then post-fixed in 1% osmium tetroxide (OsO4) for 90 minutes at room temperature and dehydrated by increasing concentration of alcohol. Following washing with propylene oxide, cells were embedded in epoxy resin. The fields of interest were selected on thin sections (0.5 μm) stained with methylene blue and safranin. Subsequently, ultrathin 60-80 nm thick sections were collected on a 300-mesh copper grid and stained with uranyl acetate and lead citrate. All reagents were purchased from Sigma Aldrich, St. Louis, MO. Ultrastructural analysis of the four experimental treatments was qualitatively performed under a transmission electron microscope (Philips EM 208S, Fei Electron Optics BV, Eindhoven, Netherlands) and high-power micrographs were collected at different magnifications.

**Periodic-Acid Schiff and May-Grunwald Giemsa Staining**

Cyto-centrifuged (Centurion Scientific, Church Farm, Stoughton Chichester, West Sussex) treated cells were stained with a Periodic Acid Schiff (PAS) kit by using a Leica ST5020 Autostainer XL (Leica Biosystems Nussloch GmbH, Germany) or with May Grunwald reagent (Merck Millipore, #1.01425.0500). Images were obtained using an optical microscope (Leica ICC50 W, Leica Microsystem, Wetzlar, Germany).

**Lysotracker detection**

PF382 and SUPT1 T-ALL cell line were exposed to G9a inhibitors for 48 hours and then pulse labeled with 75 nM of the acidotropic dye LysoTracker Red DND-99 (LTRed; Invitrogen #L7528; 1 mM stock solution in DMSO) for 90 minutes at 37°C. For immunofluorescence studies, cells were centrifuged on cytospin slides and immediately analyzed on an EVOS FL fluorescence microscope (Thermo scientific, Waltham MA, USA) with a filter set at 590 nm of fluorescence emission.

**Immunofluorescence**

Cells were spotted on immunofluorescence slides by a cytospin centrifuge (CR2000 Small Prime Centrifuge, Centurion) and fixed for 10 min in PBS, 4% paraformaldehyde (Thermo Fisher Scientific, Waltham MA, USA #28908) at 4°C. After 10 minutes of permeabilization in PBS, 0.4% Triton X-100 (Sigma-Aldrich, St. Louis, MO, USA, #T-9284) at room temperature, cells were blocked in PBS, 5% bovine serum albumin, 0.1% Triton X-100 and 1% goat serum (Abcam, #ab138478) for 1 hour at room temperature. The mouse monoclonal antibody against H3K9me2 (Abcam, Cambridge, United Kingdom, #ab1220) and the rabbit monoclonal antibody against G9a/*EHMT2* (Cell Signaling Technology, Danvers, MA, USA, #33065) were diluted in blocking solution and cells were incubated for 1 hour at room temperature. Alexa Fluor 488 (Invitrogen, Carlsbad, CA, USA, #A11029) and Alexa Fluor 568 (Invitrogen, Carlsbad, CA, USA, #A11036) were used as secondary antibodies and cells were stained 1 hour at room temperature protected by the light. Nuclei were stained with DAPI (Sigma-Aldrich, #D9542). Coverslips were mounted in Prolong Gold Antifade reagent (Thermo Fisher Scientific, Waltham MA, USA #P36934). Images were captured using an EVOS FL microscope (Thermo Fisher Scientific, Waltham MA, USA) and analyzed using ImageJ software (http://rsbweb.nih.gov/ij/).

**Seahorse XFp Glycolytic Rate assay**

The glycolysis in live cells was measured with the Seahorse XFp Glycolytic Rate Assay (Agilent Technologies, Santa Clara, CA, USA, #103346-100) and with a Seahorse XFp Analyzer (Seahorse Bioscience, North Billerica, MA) that directly measures real time extracellular acidification rates (ECAR) and oxygen consumption rate (OCR). On the day of analysis cells were plated at 300,000 cells/well and cultured in the XFp cell culture microplate with the Seahorse XFp RPMI medium without phenol red (#103336-100) supplemented with 10 mmol/L glucose, 1 mmol/L sodium pyruvate, 2 mmol/L glutamine, 5 mM HEPES. Cells were then incubated at 37°C in a non-CO_2_ incubator for 1 hour. The entire warm medium was adjusted to pH 7.4 on the day of the assay. The initial 35 min reading recorded the basal glycolytic rate over 3 measurement periods. Next, two subsequent injections followed, comprising 0.5 μM Rotenone/ Antimycin A, Rot/AA (inhibitors of mitochondrial electron transport chain) to inhibit mitochondrial oxygen consumption (and therefore CO^2^- derived protons), and 50 mM 2-deoxy-D-glucose (2-DG), a glucose analog which inhibits glycolysis. The assay data were automatically recorded and calculated by the Seahorse XFp software.

**Histone PTM Quantification**

PF382 and SUPT1 cells were treated with G9a inhibitors for 48 hours. Cells were counted with Trypan blue (mixed 1:1 with 50 µl of cells). For each cell line, one of the two samples were subjected to a two-step lysis procedure in which the cell pellet was suspended in a hypotonic buffer containing Nonidet NP-40 for 30 min on ice. Nuclei were pelleted, and acid extraction was performed for two hours at 4°C. The second sample was used in a one-step lysis procedure in which the cell pellet is directly suspended in the acid extraction buffer and incubated for two hours at 4°C. Cellular debris was pelleted, and lysate aliquots were frozen in a methanol-dye ice bath and stored at negative 80°C until testing. Based on higher signals, the two-step lysis method was selected for the experimental samples. Histone levels were determined by a five point 2.5-dilution series of the samples using the H3 Total bead. Multiplex assays were performed using sample volumes normalized for H3 concentration. Beads were added to wells in 25 µl Assay Buffer supplemented with Inhibitor Cocktails (ABIC) for proteases, phosphatases and HDACs. Samples as a three-point 1.4 dilution series were added to wells in 25 µl in duplicate and incubated for 1 hour at room temperature. Three 100µl washes with 1X wash buffer (PBS containing 0.05% Tween-20) were performed using plate magnet to retain beads. 50 µl biotinylated Histone H3 antibody diluted 1:500 in assay buffer was added for the high abundance PTM multiplex assay for 1 hour with agitation. Reporter concentration for the low abundance PTM multiplex was increased twofold (1:250 dilution). Fifty µl of SAPE diluted 1:100 in assay buffer was added to each well and incubated for 30 min with agitation. Beads were collected on a plate magnet and the SAPE solution discarded. The assay plate was removed from the plate magnet and beads suspended in 100 µl 1X wash buffer and read on the Luminex MagPix instrument.

**RT-PCR, RNA-Seq**

RNA was extracted from PF382 cells using the RNAeasy Mini Kit as per the manufacturer’s protocol (Qiagen, Hilderberg, Germany #740104). Sample quality was assessed using an agilent 2100 BioAnalyzer (Santa Clara, CA, USA). Sample libraries were prepared using NEBNext® UltraTM RNA Library Prep Kit for Illumina® (NEB, USA) following manufacturer’s recommendations and index codes were added to attribute sequences to each sample and sequenced on Illumina HiSeq with paired-end 125bp/150bp to achieve an average of 40 M reads per sample at Novogene (Beijing, China). The reads were aligned to the GRCh37/hg19 human genes, and quality control tests for the aligned reads and for replicate consistency were performed by using by the Phred score algorithm. Gene level reads and gene level expression estimate were computed as FPKM which takes into account the effects of both sequencing depth and gene length on counting of fragments^1^. Differential expression between the control (DMSO) and treatment (sgRNA or UNC0642) mRNA transcripts were calculated with the union mode at HTSeq software (Novogene (Beijing, China) ranked according to signal to noise ratio (snr) (*P*<0.05 and Log_2_ fold changes > 1.5). The GSEA v4.0.3 and transcription factor enrichment analysis TF ChEA3 softwares were used for enrichment studies. The RNA-Seq data for this study will be available for download from the Gene Expression Omnibus (GEO) repository upon manuscript publication.

**Preclinical validation experiments in 3D cell culture**

3D bioreactors, VITVO, (Rigenerand S.r.l., Medolla, Modena, Italy) were primed with 2 ml of cell culture medium to ensure a complete wetting of the 3D matrix and then loaded with 1.5x10^6^ PF382 or SUPT1 cells suspended in 1.2 ml of cell medium by a 5 ml syringe (Becton Dickinson and Co, Franklin Lakes, NJ, USA). Cells were treated 24 hours after loading at the indicated drugs concentrations. Real Time-GLO™ MT substrate was added in the medium following manufacturer instruction and luminescence was measured 2 hours after. This process has been repeated at day zero, one, two and three days after treatment and, luminescence quantified by a Victor X4 multi plate readers (Perkin Elmer, Waltham, MA, USA). Live/Dead^®^ (Thermo Fisher Scientific, Waltham MA, USA #L3224) and Lysotracker (LTRed; Invitrogen #L7528) assays were performed three days after treatment following manufacturer instruction. 3D co-culture model of T-ALL cell lines was established growing PF382 and SUPT1 cell lines on HS-5 bone marrow stromal cell line. 0.15x10^6^ HS-5 cells were re-suspended in 1.2 ml of DMEM supplemented with 5ul/ml Hoechst (Thermo Fisher Scientific, Waltham MA, USA NucBlue™ Live ReadyProbes™ Reagent #R37605) and loaded into the VITVO inner chamber. After 24 hours devices were washed out carefully to remove Hoechst dye from the 3D cell culture and 1.5x10^6^ PF382 or SUPT1 in 1.2 ml of RPMI were loaded on HS-5 colonized scaffolds. Before treatment we waited additional 24 hours to obtain cell-cell stable interactions between stromal and T-ALL cell lines. Then, mixed cell cultures where treated with DMSO or UNC0642 at the indicated concentrations and imaged before and 3 days after treatment for green fluorescent protein (GFP) quantification analysis. Imaging was carried out avoiding field of views with high autofluorescence of the scaffold’s matrix and air bubbles. Images were captured by EVOS FL microscope (Thermo Fisher Scientific, Waltham MA, USA) using Olympus long distance 4x objective and analyzed using ImageJ software (http://rsbweb.nih.gov/ij/).

**T-ALL *in Vivo* Studies**

Non-obese diabetic, severe combined immune-deficient, interleukin (IL)-2 receptor gamma-deficient mice (NSG) were maintained in pathogen-free facilities at the University of Perugia. Local IACUC approved all procedures. MOLT16 T-ALL cells [2.5 x 10^6^ cells in 250 µL of phosphate buffer saline (PBS)] were injected into the retro-orbital venous sinus in adult (10-12 weeks old) non-irradiated NSG mice (day of transplant was counted as day 0). *In vivo* treatment started at day 10 after transplant. Mice received UNC0642 at a dose of 5 mg/kg every 48 hours, for 12 days by intraperitoneal injection. Two mice died prematurely due to drug toxicity, leaving 7 evaluable mice in the UNC0642 treated arm and 5 in the vehicle arm. To assess CD45+ infiltration in the bone marrow and the spleen, we applied a grid to each IHC image. The total area, equal to 4608 squares, was set at 100. The number of red squares, equivalent to CD45 positive cells (human MOLT-16 infiltrating cells), has been manually counted by an expert pathologist and reported as a percentage. Multiple fields from three representative mice per group were included for the study. 4 x 10^6^ patient derived leukemia xenograft (PDLX) T-ALLs cell, from previously established models, were injected into the retro-orbital venous sinus in adult (12-16 weeks old) sublethally-irradiated (0.9 Gy) NSG mice, 6 mice per group, males and females equally distributed among vehicle and treated arms.

PDLX-0121 (slowly grower), *TCRB*-*MYC*/t(7;8)(q34;q24) rearrangement, *CDKN2AB*/9p21 biallelic deletion, *PTEN*/10q24 deletion, received 26 doses of UNC0642 at 5 mg/kg every 48 hours starting 5 days post-transplant (euthanized 70 days post-transplant). PDLX-0122 (fast grower), *TRB*-*HOXA*/inv(7)(p15q34), *PTPN2*/18p11 deletion, *TP53*/17p13 deletion, *MYC*-translocation/t(8;?)(q24;?), received for 9 doses UNC0642 at 5 mg/kg every 48 hours starting 1-day post-transplant (euthanized 20 days post-transplant). The control groups received 10% DMSO (Carlo Erba Reagents S.r.l.) and 90% corn oil (Sigma-Aldrich).

Paraformaldehydes fixed organs were prepared for standard hematoxylin and eosin staining and for immunohistochemistry using human CD45 antibody (clones 2B11 + PD7/26 Dako, Agilent) and H3K9me2 (Abcam, ab#1220). Images were acquired using an Olympus BX-51 microscope (Olympus Life Science) and DM500 (Leica).

The antileukemic activity of UNC0642 in PDLX (#0122) was assessed by a survival study, allowed by the short-term engraftment and immediate development of leukemia (Kaplan-Meier method), and by measuring human CD45 expression (clone HI30, BD Biosciences, New Jersey, USA) on bone marrow and peripheral blood by flow cytometry (FACS CANTO, BD Biosciences, San Jose, CA, USA)

CD1 mice were bred in the Animal Facility of the University of Parma and housed in a temperature-controlled (20–24 °C) room, under 12h light/12h dark cycle. Animal procedures were performed in accordance with European Community Directive 2010/63/UE and approved by the Ethics Committee of the University of Parma (Prot. 51/OPBA/16). 8 female mice aged between 4-6 weeks were used for the experiments. After cervical dislocation, the thymus was immediately excised, placed in a 6-well plate (Corning TM) containing 5 ml RPMI-1640 (Thermo Fisher Scientific, #MT10040CV), macroscopically separated from surrounding fat and connective tissue, and immediately used for thymocytes isolation.

**Statistical Analysis and Image processing**

Cell proliferation and viability data were analyzed using Graphpad PRISM software version 7 (La Jolla, CA, USA) and the Microsoft Excel Package, 2014 version (Redmond, WAS, USA). Flow cytometry data were analyzed with FlowJo analytical software version 6 or 7 (Tree Star LLC, Ashland, OR, USA).

**REFERENCES**

1. Trapnell C, Williams BA, Pertea G, Mortazavi A, Kwan G, van Baren MJ*, et al.* Transcript assembly and quantification by RNA-Seq reveals unannotated transcripts and isoform switching during cell differentiation. *Nat Biotechnol* 2010 May; **28**(5)**:** 511-515.
