# Supplementary material for: Identification of an Epi-metabolic dependency on EHMT2/G9a in T-cell acute lymphoblastic leukemia
Source: Cell Death Dis. 2022 Jun 17;13(6):551. doi: 10.1038/s41419-022-05002-5 (PMC9203761; doi:10.1038/s41419-022-05002-5)
Supplement: Supplementary file 4 — Supplementary western blot original file [file 41419_2022_5002_MOESM4_ESM.pdf]

Figure 1G_actin

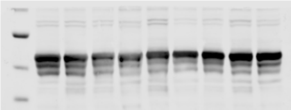

Figure 1G_ICN1

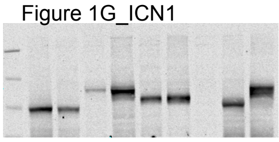

Figure 1G_EHMT1

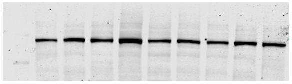

Figure 1G_G9a l

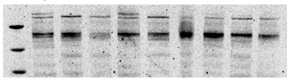

Figure 1G_G9a s

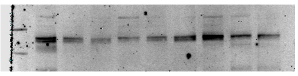

Figure 2B_PF382_G9a

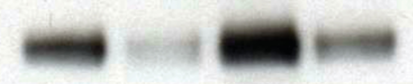

Figure 2B_SUPT1_G9a

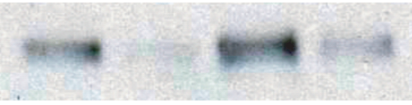

Figure 2B_SUPT1_GAPDH

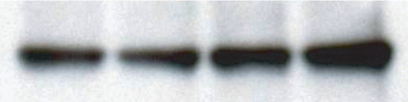

Figure 2B_PF382_GAPDH

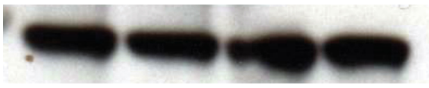

Figure 3A_H3K9me1

Figure 3A_H3K9me2

Figure 3A_H3K9me3

Figure 3A_H3

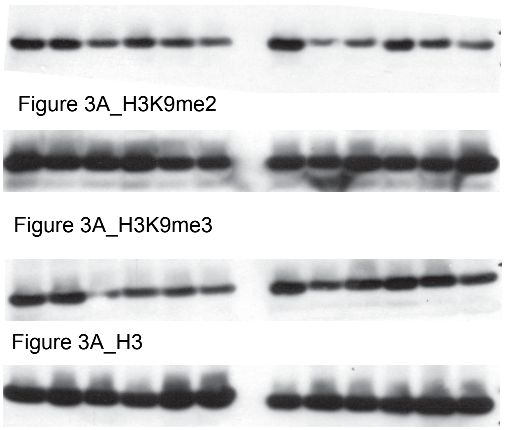

Figure 3D_H3_BIX01294_PF382

Figure 3D_H3_UNC0638_PF382

Figure 3D_H3_UNC0642_PF382

Figure 3D_H3_BIX01294_SUPT1

Figure 3D_H3_UNC0638_SUPT1

Figure 3D_H3_UNC0642_SUPT1

Figure 3D_H3K9me2_BIX01294_PF382

Figure 3D_H3K9me2_UNC0638_PF382

Figure 3D_H3K9me2_UNC0642_PF382

Figure 3D_H3K9me2_BIX01294_SUPT1

Figure 3D_H3K9me2_UNC0638_SUPT1

Figure 3D_H3K9me2_UNC0642_SUPT1

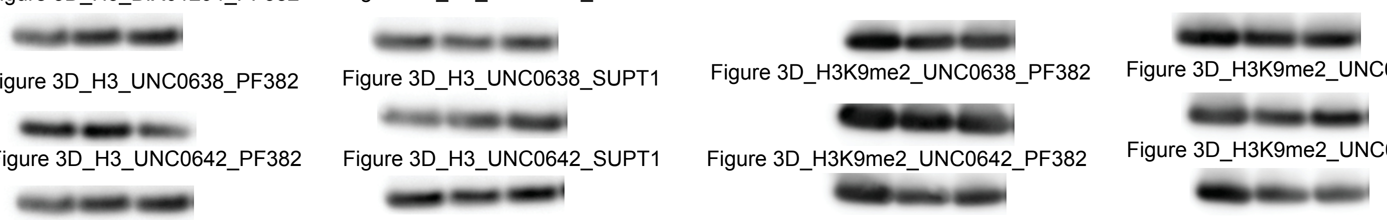

Figure 4C_SESN2

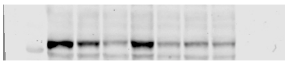

Figure 4C_Actin

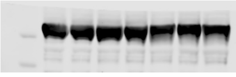

Figure 4D_PF382_G9a

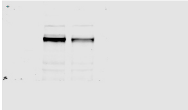

Figure 4D_PF382_HSP90

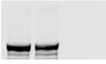

Figure 4D_PF382_SESN2

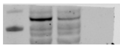

Figure 4D_PF382_H3K9me2

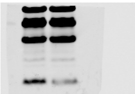

Figure 4D_PF382_H3Total

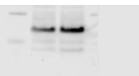

Figure 4D_SUPT1_G9a

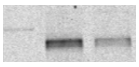

Figure 4D_SUPT1_HSP90

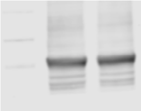

Figure 4D_SUPT1_SESN2

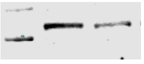

Figure 4D_SUPT1_H3K9me2

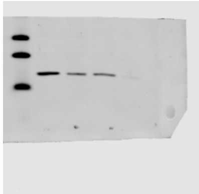

Figure 4D_SUPT1_H3Total

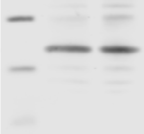

Figure 5G_PF382_GSK3A

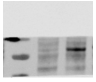

Figure 5G_SUPT1_GSK3A

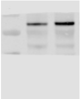

Figure 5G_PF382_GSK3B

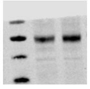

Figure 5G_SUPT1_GSK3B

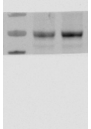

Figure 5G_PF382_GSK3

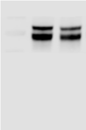

Figure 5G_SUPT1_GSK3

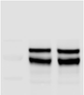

Figure 5H_PF382_GSK3A

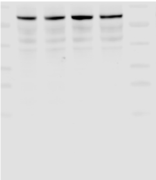

Figure 5H_PF382_GSK3B

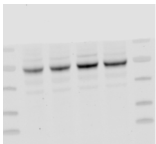

Figure 5H_PF382_GSK3

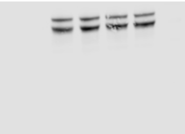

Figure 5H_PF382_HSP90

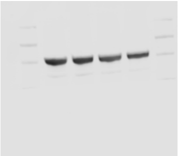

Figure 5H_SUPT1_GSK3A

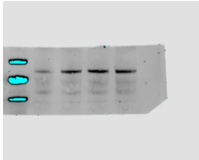

Figure 5H_SUPT1_GSK3B

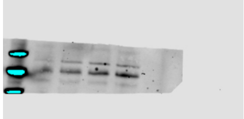

Figure 5H_SUPT1_GSK3

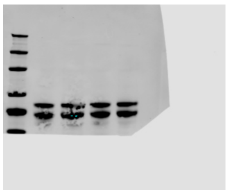

Figure 5H_SUPT1_HSP90

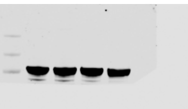

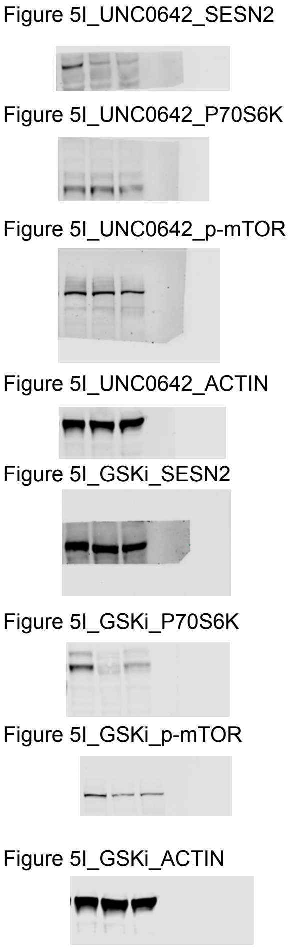
Figure 5I_UNC0642_SESN2
Figure 5I_UNC0642_P70S6K
Figure 5I_UNC0642_p-mTOR
Figure 5I_UNC0642_ACTIN
Figure 5I_GSKi_SESN2
Figure 5I_GSKi_P70S6K
Figure 5I_GSKi_p-mTOR
Figure 5I_GSKi_ACTIN

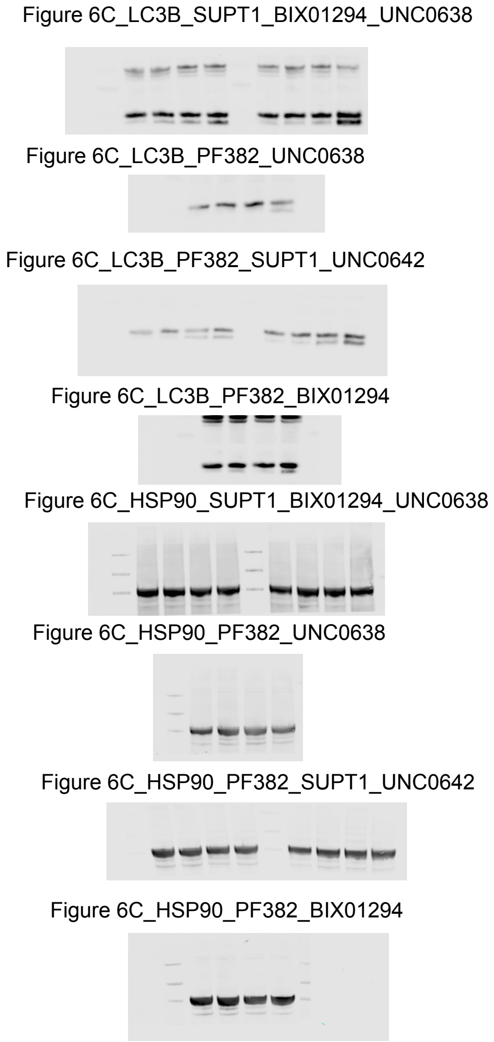
Figure 6C_LC3B_SUPT1_BIX01294_UNC0638
Figure 6C_LC3B_PF382_UNC0638
Figure 6C_LC3B_PF382_SUPT1_UNC0642
Figure 6C_LC3B_PF382_BIX01294
Figure 6C_HSP90_SUPT1_BIX01294_UNC0638
Figure 6C_HSP90_PF382_UNC0638
Figure 6C_HSP90_PF382_SUPT1_UNC0642
Figure 6C_HSP90_PF382_BIX01294

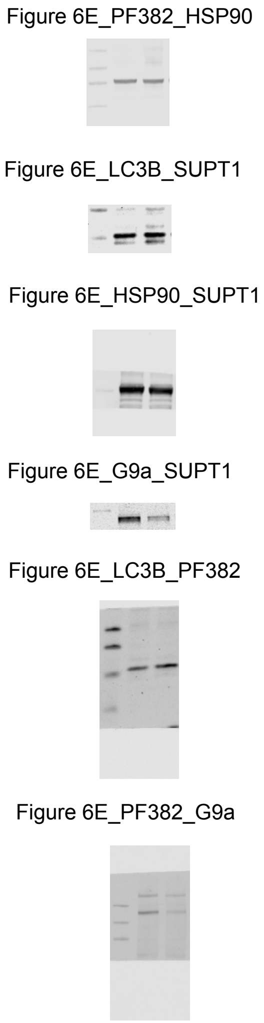
Figure 6E_PF382_HSP90
Figure 6E_LC3B_SUPT1
Figure 6E_HSP90_SUPT1
Figure 6E_G9a_SUPT1
Figure 6E_LC3B_PF382
Figure 6E_PF382_G9a

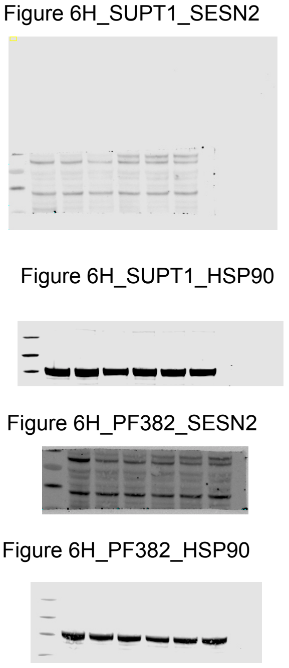
Figure 6H_SUPT1_SESN2
Figure 6H_SUPT1_HSP90
Figure 6H_PF382_SESN2
Figure 6H_PF382_HSP90

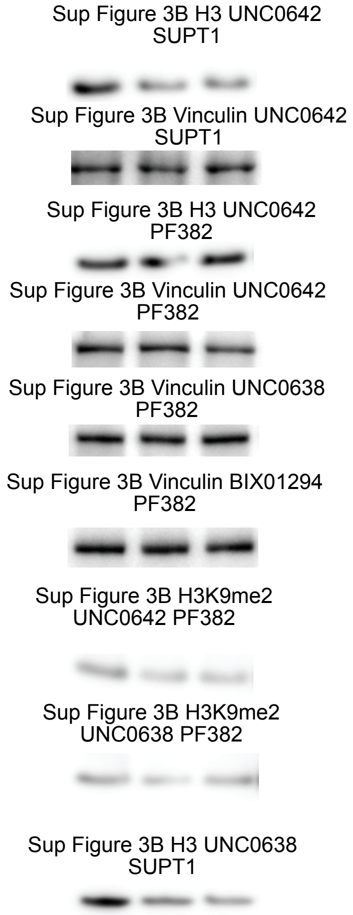
Sup Figure 3B H3 UNC0642 SUPT1
Sup Figure 3B Vinculin UNC0642 SUPT1
Sup Figure 3B H3 UNC0642 PF382
Sup Figure 3B Vinculin UNC0642 PF382
Sup Figure 3B Vinculin UNC0638 PF382
Sup Figure 3B Vinculin BIX01294 PF382
Sup Figure 3B H3K9me2 UNC0642 PF382
Sup Figure 3B H3K9me2 UNC0638 PF382
Sup Figure 3B H3 UNC0638 SUPT1

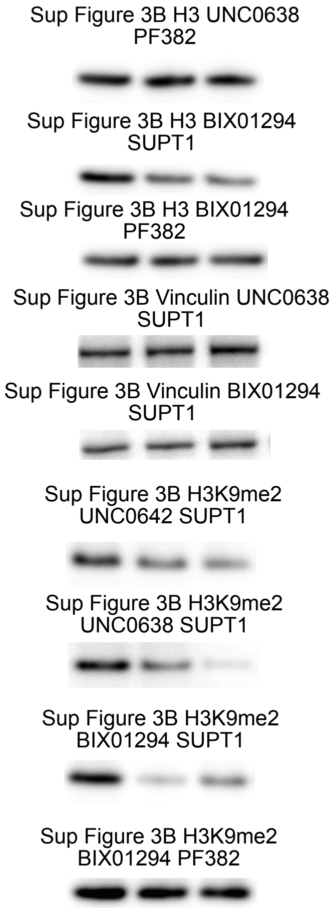
Sup Figure 3B H3 UNC0638 PF382
Sup Figure 3B H3 BIX01294 SUPT1
Sup Figure 3B H3 BIX01294 PF382
Sup Figure 3B Vinculin UNC0638 SUPT1
Sup Figure 3B Vinculin BIX01294 SUPT1
Sup Figure 3B H3K9me2 UNC0642 SUPT1
Sup Figure 3B H3K9me2 UNC0638 SUPT1
Sup Figure 3B H3K9me2 BIX01294 SUPT1
Sup Figure 3B H3K9me2 BIX01294 PF382

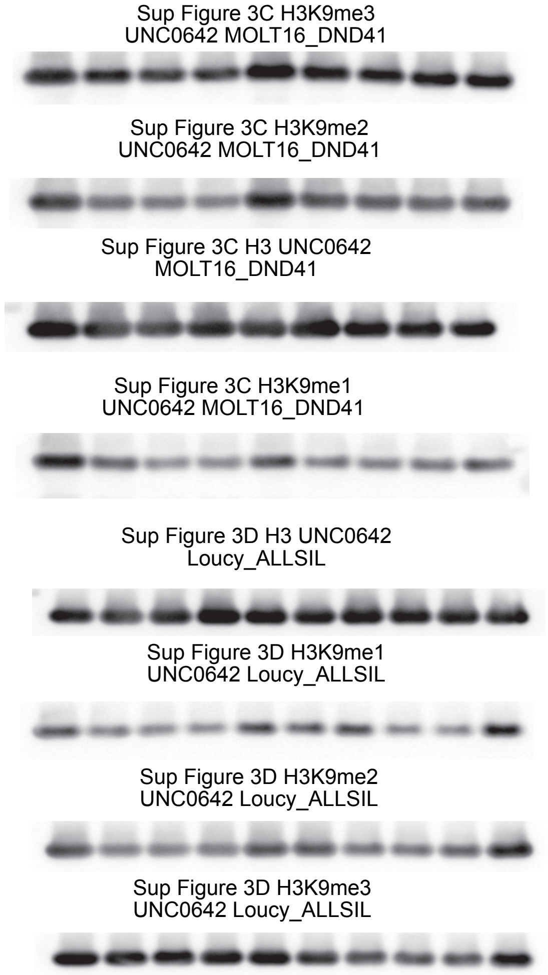
Sup Figure 3C H3K9me3 UNC0642 MOLT16_DND41
Sup Figure 3C H3K9me2 UNC0642 MOLT16_DND41
Sup Figure 3C H3 UNC0642 MOLT16_DND41
Sup Figure 3C H3K9me1 UNC0642 MOLT16_DND41
Sup Figure 3D H3 UNC0642 Loucy_ALLSIL
Sup Figure 3D H3K9me1 UNC0642 Loucy_ALLSIL
Sup Figure 3D H3K9me2 UNC0642 Loucy_ALLSIL
Sup Figure 3D H3K9me3 UNC0642 Loucy_ALLSIL

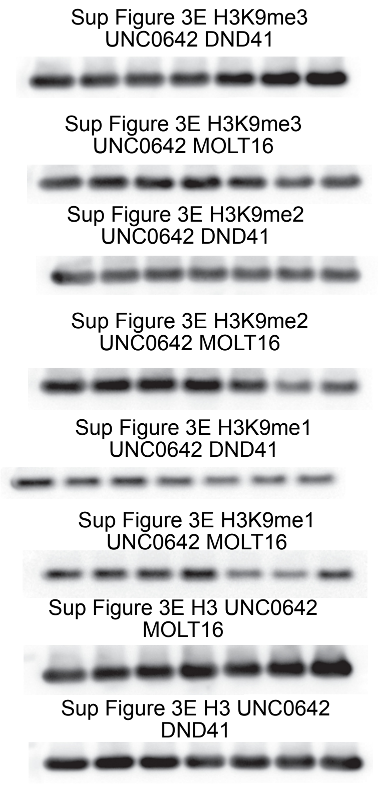
Sup Figure 3E H3K9me3 UNC0642 DND41
Sup Figure 3E H3K9me3 UNC0642 MOLT16
Sup Figure 3E H3K9me2 UNC0642 DND41
Sup Figure 3E H3K9me2 UNC0642 MOLT16
Sup Figure 3E H3K9me1 UNC0642 DND41
Sup Figure 3E H3K9me1 UNC0642 MOLT16
Sup Figure 3E H3 UNC0642 MOLT16
Sup Figure 3E H3 UNC0642 DND41

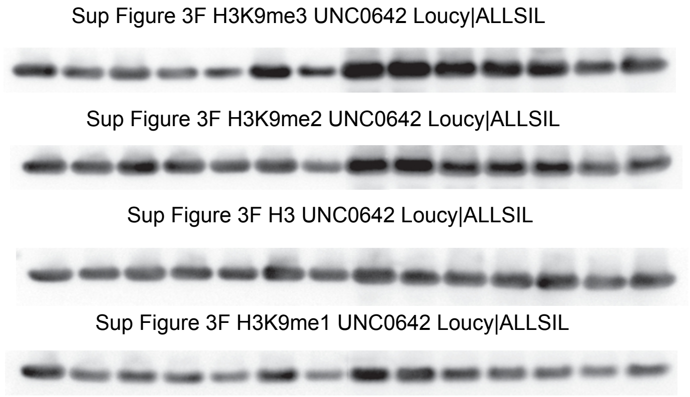
Sup Figure 3F H3K9me3 UNC0642 Loucy|ALLSIL
Sup Figure 3F H3K9me2 UNC0642 Loucy|ALLSIL
Sup Figure 3F H3 UNC0642 Loucy|ALLSIL
Sup Figure 3F H3K9me1 UNC0642 Loucy|ALLSIL

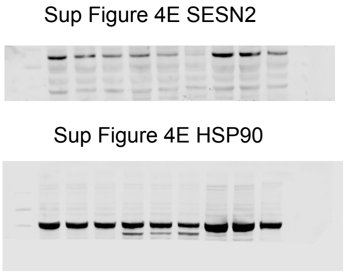
Sup Figure 4E SESN2
Sup Figure 4E HSP90

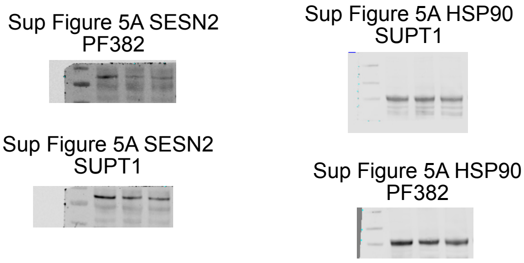
Sup Figure 5A SESN2 PF382
Sup Figure 5A HSP90 SUPT1
Sup Figure 5A SESN2 SUPT1
Sup Figure 5A HSP90 PF382

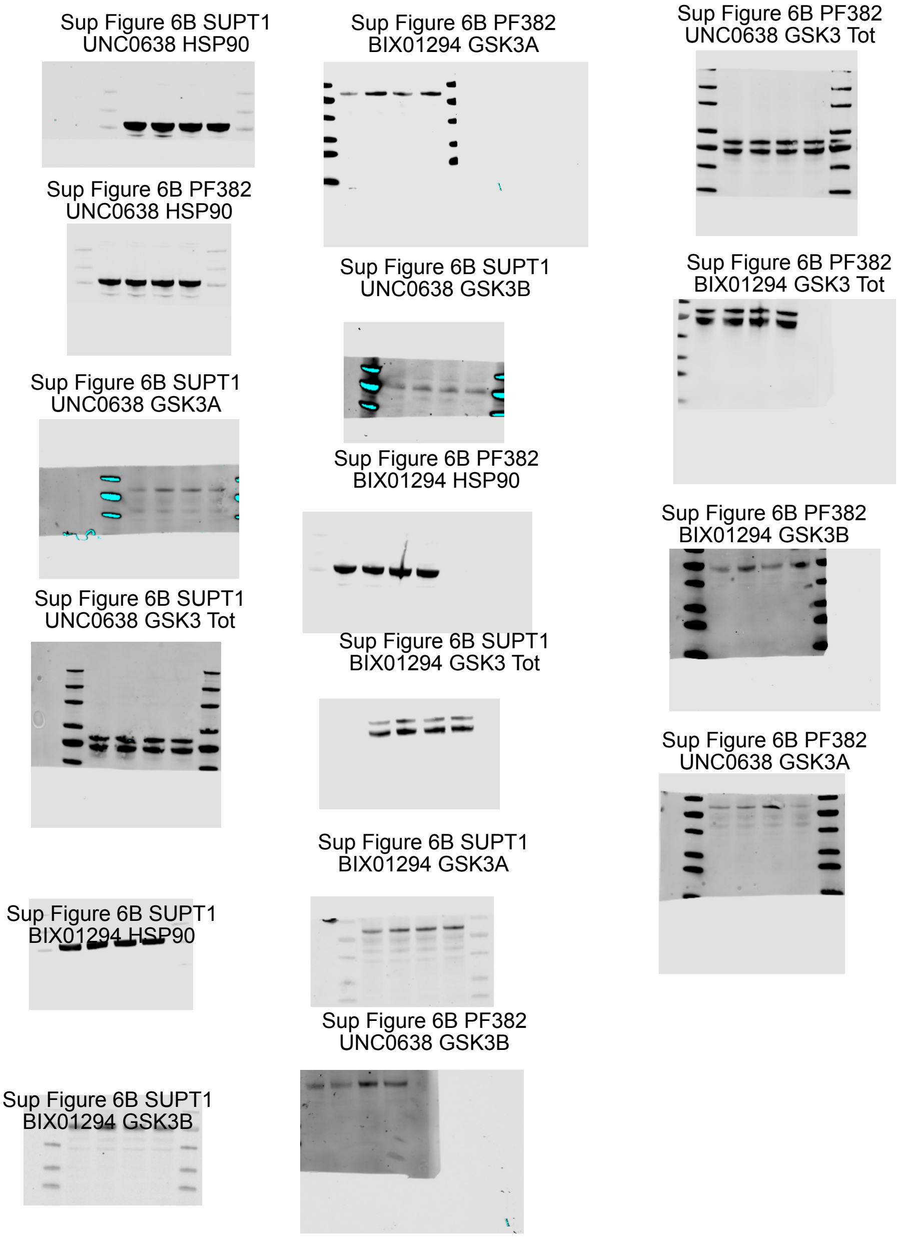
Sup Figure 6B SUPT1 UNC0638 HSP90
Sup Figure 6B PF382 BIX01294 GSK3A
Sup Figure 6B PF382 UNC0638 GSK3 Tot
Sup Figure 6B PF382 UNC0638 HSP90
Sup Figure 6B SUPT1 UNC0638 GSK3B
Sup Figure 6B PF382 BIX01294 GSK3 Tot
Sup Figure 6B SUPT1 UNC0638 GSK3A
Sup Figure 6B PF382 BIX01294 HSP90
Sup Figure 6B PF382 BIX01294 GSK3B
Sup Figure 6B SUPT1 UNC0638 GSK3 Tot
Sup Figure 6B SUPT1 BIX01294 GSK3 Tot
Sup Figure 6B PF382 UNC0638 GSK3A
Sup Figure 6B SUPT1 BIX01294 GSK3A
Sup Figure 6B SUPT1 BIX01294 HSP90
Sup Figure 6B PF382 UNC0638 GSK3B
Sup Figure 6B SUPT1 BIX01294 GSK3B

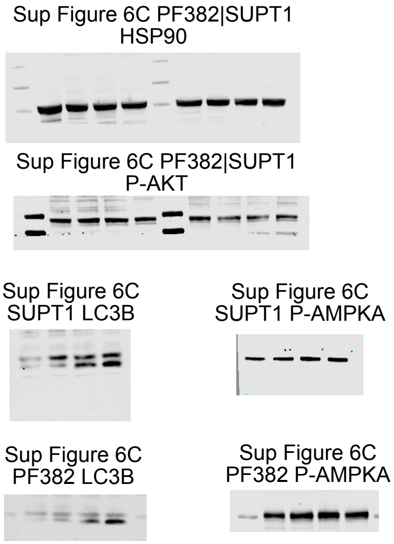
Sup Figure 6C PF382|SUPT1 HSP90
Sup Figure 6C PF382|SUPT1 P-AKT
Sup Figure 6C SUPT1 LC3B
Sup Figure 6C SUPT1 P-AMPKA
Sup Figure 6C PF382 LC3B
Sup Figure 6C PF382 P-AMPKA

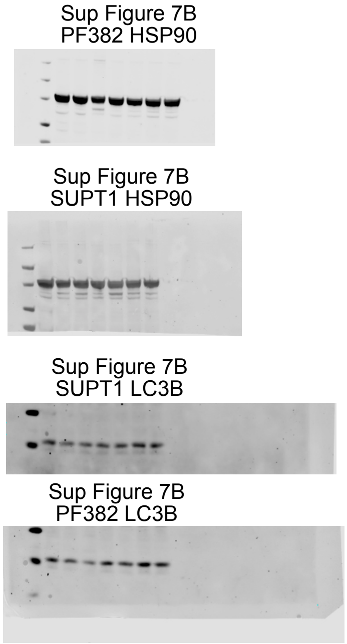
Sup Figure 7B PF382 HSP90
Sup Figure 7B SUPT1 HSP90
Sup Figure 7B SUPT1 LC3B
Sup Figure 7B PF382 LC3B
